# A Chemical-Genomic Screen of Neglected Antibiotics Reveals Illicit Transport of Kasugamycin and Blasticidin S

**DOI:** 10.1371/journal.pgen.1006124

**Published:** 2016-06-29

**Authors:** Anthony L. Shiver, Hendrik Osadnik, George Kritikos, Bo Li, Nevan Krogan, Athanasios Typas, Carol A. Gross

**Affiliations:** 1 Graduate Group in Biophysics, University of California, San Francisco, San Francisco, California, United States of America; 2 Department of Microbiology and Immunology, University of California, San Francisco, San Francisco, California, United States of America; 3 European Molecular Biology Laboratory, Genome Biology Unit, Heidelberg, Germany; 4 Department of Chemistry, University of North Carolina-Chapel Hill, Chapel Hill, North Carolina, United States of America; 5 QB3, California Institute for Quantitative Biosciences, San Francisco, California, United States of America; 6 Department of Cellular and Molecular Pharmacology, University of California, San Francisco, San Francisco, California, United States of America; 7 Gladstone Institutes, San Francisco, California, United States of America; 8 Department of Cell and Tissue Biology, University of California, San Francisco, California, United States of America; Uppsala University, SWEDEN

## Abstract

Fighting antibiotic resistance requires a deeper understanding of the genetic factors that determine the antibiotic susceptibility of bacteria. Here we describe a chemical-genomic screen in *Escherichia coli* K-12 that was designed to discover new aspects of antibiotic resistance by focusing on a set of 26 antibiotics and other stresses with poorly characterized mode-of-action and determinants of resistance. We show that the screen identifies new resistance determinants for these antibiotics including a common signature from two antimicrobials, kasugamycin and blasticidin S, used to treat crop diseases like rice blast and fire blight. Following this signature, we further investigated the mechanistic basis for susceptibility to kasugamycin and blasticidin S in *E*. *coli* using both genetic and biochemical approaches. We provide evidence that these compounds hijack an overlapping set of peptide ABC-importers to enter the bacterial cell. Loss of uptake may be an underappreciated mechanism for the development of kasugamycin resistance in bacterial plant pathogens.

## Author Summary

Bacterial species differ in their susceptibility to antibiotics but the reason for these differences remains an open question. Understanding the genetic basis of antibiotic susceptibility will be critical for predicting the efficacy of new antibiotics and possibly finding new antibiotic targets. Here we report a large-scale study that connects bacterial genes to antibiotics, using a set of antibiotics that were chosen to include poorly characterized compounds. We discovered genes that confer resistance to a number of neglected antibiotics, expanding our knowledge of gene function and antibiotic resistance in *Escherichia coli* K-12. Starting from this large-scale screen, we then investigated how two antibiotics with a common history, kasugamycin and blasticidin S, enter bacterial cells. Both mimic naturally occurring nutrients to trick *E*. *coli* into actively bringing them inside. Kasugamycin is used to control microbes that cause agricultural diseases and mutations that reduce uptake like those we describe here may be an underappreciated factor in the development of resistance to kasugamycin.

## Introduction

The emerging threat of antibiotic resistance [1] necessitates new efforts and ideas to control bacterial pathogens. Mapping the determinants of antibiotic resistance in bacteria will be critical for evaluating new antibiotics. In addition to the direct target of the antibiotic, drug efflux, drug permeability, and stress response pathways all contribute to resistance [2]. Global genetic approaches such as chemical-genomic screens, which measure the sensitivities of a large library of mutants to a set of stresses, can be a first-step in discovering resistance determinants and characterizing the mode-of-action of antibiotics. Chemical-genomic screens in the model bacterium *Escherichia coli* K-12 [3–10] have already provided a critical resource for the bacterial research community and catalyzed insights into molecular systems critical for bacterial viability, stress survival, and resistance to antibiotics [11–15].

Despite these successes, the chemical-genetic space of *E*. *coli* remains largely unexplored, as only slightly more than 50% of the genes in *E*. *coli* K-12 are “responsive”, defined as having a statistically significant fitness effect for at least one stress [8]. Reasoning that the remaining unresponsive genes may encode resistance determinants to previously untested antibiotics and stresses, we conducted a new chemical-genomic screen of the previously screened library [8,10] focusing on antibiotics with unique or unknown modes of action. We integrated the data from this screen with the results of Nichols et al. [8] to create an expanded chemical-genomics dataset that revealed new determinants of antibiotic resistance.

From this dataset, we further investigated resistance to the antibiotics kasugamycin (Ksg) [16] and blasticidin S (BcS) [17]. Both antibiotics were discovered in the mid-20^th^ century as antifungals effective against *Magnaporthe oryzae*, the causative agent of rice blast. Kasugamycin has a continuing use in the treatment of *M*. *oryzae*, bacterial pathogens of rice, and *Erwinia amylovora*, the bacterial pathogen that causes fire blight. We discovered that both antibiotics enter bacterial cells using illicit transport, the active uptake of non-physiological compounds, through two peptide ABC-importers. We suggest that loss-of-function mutations in homologous peptide ABC-importers are likely to play a role in the development of kasugamycin resistance for *E*. *amylovora* and many other pathogens.

## Results

### The chemical-genomic screen substantially expands known connections in *E*. *coli*

We tested the sensitivities of 3975 mutants of *E*. *coli* K-12 to 57 stresses, split between new and previously screened conditions. The new stresses included neglected antibacterial compounds and other noxious chemicals with poorly characterized modes of action (Table 1). We pinned the arrayed mutant library onto agar plates containing each compound, imaged the plates after suitable colony growth, and quantified colony opacity using the image analysis software Iris [10]. We assigned fitness-scores to each mutant, using an in-house software package that built upon previous analyses [8,18] by implementing additional filtering and normalization steps to improve data quality (Methods). These fitness-scores represent the statistical significance of a change in colony size for a particular condition, with negative and positive fitness-scores representing sensitivity and resistance, respectively.

**Table 1:** New stresses in the chemical genomic screen.

Stress	Drug Family/ Stress Type	Biological Target
D,L-serine hydroxamate	hydroxamic acid	seryl-tRNA synthetase
pseudomonic acid A	monoxycarbolic acid	isoleucyl-tRNA synthetase
blasticidin S	aminonucleoside	ribosome
kasugamycin	inositol-based aminoglycoside	ribosome
clindamycin	lincosamide	ribosome
10°C growth	cold shock	multiple targets
cinoxacin	quinolone	gyrase
chlorhexidine	bisguanide	cell membrane
sodium fluoride	anion	enolase
5-fluorouridine	uracil analogue	thymidylate synthase
gliotoxin	epidithiodiketopiperazine	unknown
holomycin	dithiolopyrrolone	unknown
thiolutin	dithiolopyrrolone	unknown
silver(II) nitrate	divalent cation	unknown
azelaic acid	dicarboxylic acid	unknown
isopropanol	alcohol	unknown
n-butanol	alcohol	unknown
t-butanol	alcohol	unknown
phenol	alcohol	unknown
guanidine	chaotrope	unknown
urea	chaotrope	unknown
DMSO	polar organic solvent	unknown
5-methylanthranilic acid	tryptophan precursor analogue	unknown
5-methyltryptophan	tryptophan analogue	unknown
7-azatryptophan	tryptophan analogue	unknown
4°C survival	cold shock	unknown

**Fig 1:**
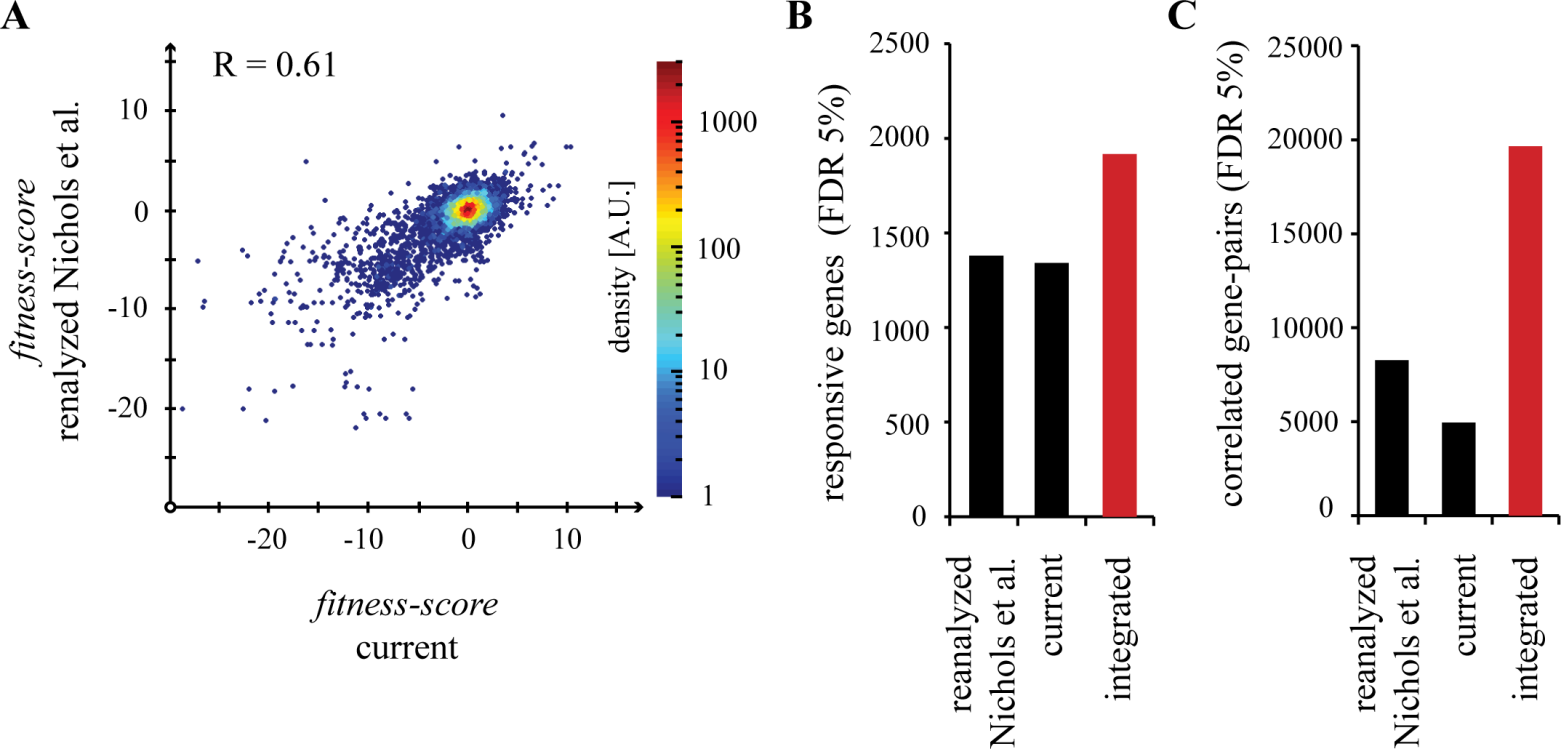
The chemical genomic screen expands our view of gene function and intrinsic resistance in *E*. *coli* K-12. (A) A scatter plot of individual fitness-scores for conditions present in both screens (n = 17). Measurements between screens are reproducible, with a Pearson’s correlation of 0.61. (B) Conditional-phenotypes were assigned using a stress-specific cutoff for fitness-scores that allowed a false discovery rate (FDR) of 5%. A responsive gene is defined as a gene with at least one phenotype in the dataset. (C) Significant correlations between genes were determined using a cutoff for Pearson’s correlation that allowed an FDR of 5%.

After reanalyzing the original images from the Nichols et al. [8] screen with our improved workflow, we integrated both datasets. Fitness-scores from stresses present in both screens were significantly correlated (Fig 1A). A responsive gene is defined as having at least one conditional-phenotype in the dataset. We identified more than 5,000 conditional-phenotypes for the 26 new stresses, as well as more than 500 additional responsive genes from the 57 stresses tested (14% of the library) (Fig 1B). The conditional-phenotypes that identified additional responsive genes were spread evenly throughout the current screen, ranging from 5 to 54 conditional-phenotypes per condition from within the set of new responsive genes. The integrated dataset also contained more than double the number of statistically significant correlations between genes than each screen considered separately (Fig 1C).

This increase in the number of significant correlations came from two factors. First, the integrated dataset captured more conditional-phenotypes that in turn drove higher correlations for some genes. Second, smaller datasets require more stringent cutoffs for statistical significance that exclude a sizable fraction of the correlations (S1 Fig). Because of these factors, integration of chemical genomic screen with a larger dataset was critical for extracting as much information as possible from the new conditions. We use this integrated dataset (S1 Dataset) in all further analyses.

### A global picture of resistance to neglected antibiotics

**Fig 2:**
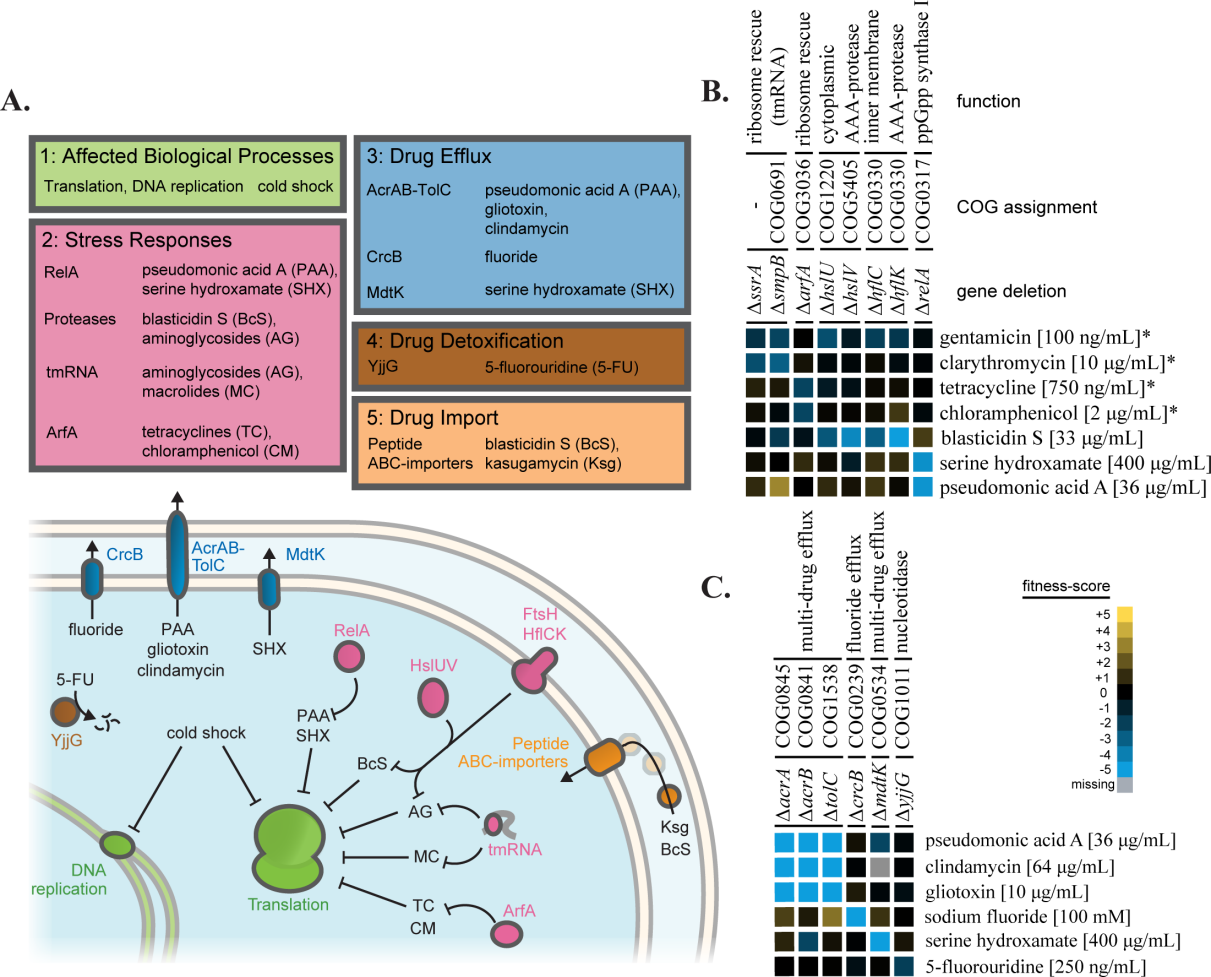
A diverse set of pathways contribute to antibiotic resistance. Fitness-scores from the integrated dataset connected antibiotic resistance to multiple biological pathways. (A) Genes with different resistance mechanisms (target pathway, stress response, drug efflux, detoxification, and drug import) that protect against stresses from the current screen are organized according to mechansim. (B) A heatmap of fitness-scores for translation related genes. Sensitivities of deletions of various translation associated factors distinguish between drug families targeting translation. Sensitivities to aminoglycosides (gentamicin), macrolides (clarithromycin), tetracyclines (tetracycline), chloramphenicol, and tRNA synthetase inhibitors (pseudomonic acid A and serine hydroxamate) are shown. Fitness-scores from the integrated dataset that were determined in Nichols et al. [8] are marked with an asterisk (*). (C) A heatmap of fitness-scores for genes related to drug efflux and detoxification. Multiple drugs from the new screen were connected with the major efflux pump of *E*. *coli*, AcrAB-TolC.

The pathways that sense and respond to different stresses are nearly as diverse as the types of stress that are encountered. Genes that are involved in drug permeability, drug efflux and degradation, stress responses, and the drug target all contribute to resistance. The chemical-genomic screen revealed a global picture of antibiotic resistance that reflected this diversity in mechanism (Fig 2A).

Growth at 10°C resulted in multiple sensitivities from genes in pathways known to be affected by cold shock, with almost double the number of cold-sensitive mutations as the next lowest temperature (16°C). 10°C-sensitive genes were most enriched for COG categories [19] related to translation (J, p = 0.005) and DNA replication and repair (L, p = 0.01), with 20% of the sensitive genes falling into one of these two categories (Fig 2A, S1 Table). Ribosome assembly and function is particularly sensitive to temperatures of 10°C and below [20], which may explain part of the expansion of sensitivities at this temperature.

Deletions in the trans-translation complex (Δ*ssrA* and Δ*smpB*) were sensitive to aminoglycosides and macrolides (Fig 2B), while the deletion of the alternative ribosome rescue factor (Δ*arfA*) was sensitized to members of the tetracycline family and chloramphenicol (Fig 2B). Deletions in the cytoplasmic protease HslUV (Δ*hslU*,*V*) and adaptors to the inner membrane protease FtsH (Δ*hflC*,*K*) were sensitized to blasticidin S (Fig 2B). These protease deletions have been demonstrated to be sensitive to aminoglycosides, both in *E*. *coli* [5,8] and in *P*. *aeruginosa* [21,22]. This set of pathways could be directly counteracting harmful effects of the translation inhibitors, with different pathways required to respond to unique mechanisms of action. As an example, deletion of the ribosome-bound ppGpp synthase (*ΔrelA*) resulted in sensitivity to both tRNA synthetase inhibitors used in the screen (Fig 2B). RelA has a known role in sensing and responding to uncharged tRNAs in the A-site of the ribosome [23].

Sensitivities from the screen also indicated that many of the antibiotics were subject to drug efflux and degradation (Fig 2A). Loss of AcrAB-TolC, the major efflux pump of *E*. *coli* [24], sensitized cells to gliotoxin, clindamycin, and pseudomonic acid A (Fig 2C). Other efflux pumps had more specific effects. The deletion strain Δ*crcB* was sensitive to fluoride [25] while Δ*mdtK* was sensitive to serine hydroxamate [26]. The strain Δ*yjjG* was sensitive to 5-fluorouridine, consistent with the role of YjjG as a protective nucleotidase [27] (Fig 2C). For Δ*crcB*, Δ*mdtK*, and Δ*yjjG* these chemical sensitivities were the strongest for each strain across the integrated dataset.

Additionally, gene deletions within two peptide ABC-importers were resistant to the antibiotics kasugamycin and blasticidin S. The ABC-importers of bacteria have been implicated in uptake of a diverse set of antibiotics, including blasticidin S, in a process termed illicit transport [28–32]. This phenotype was particularly interesting because of the agricultural importance of kasugamycin and because the uptake mechanism for these drugs has not been described in *E*. *coli* K-12. We therefore tested whether Ksg and BcS were directly imported by these peptide ABC-importers.

### Two peptide ABC-importers determine the susceptibility of E. coli K-12 to kasugamycin and blasticidin S

**Fig 3:**
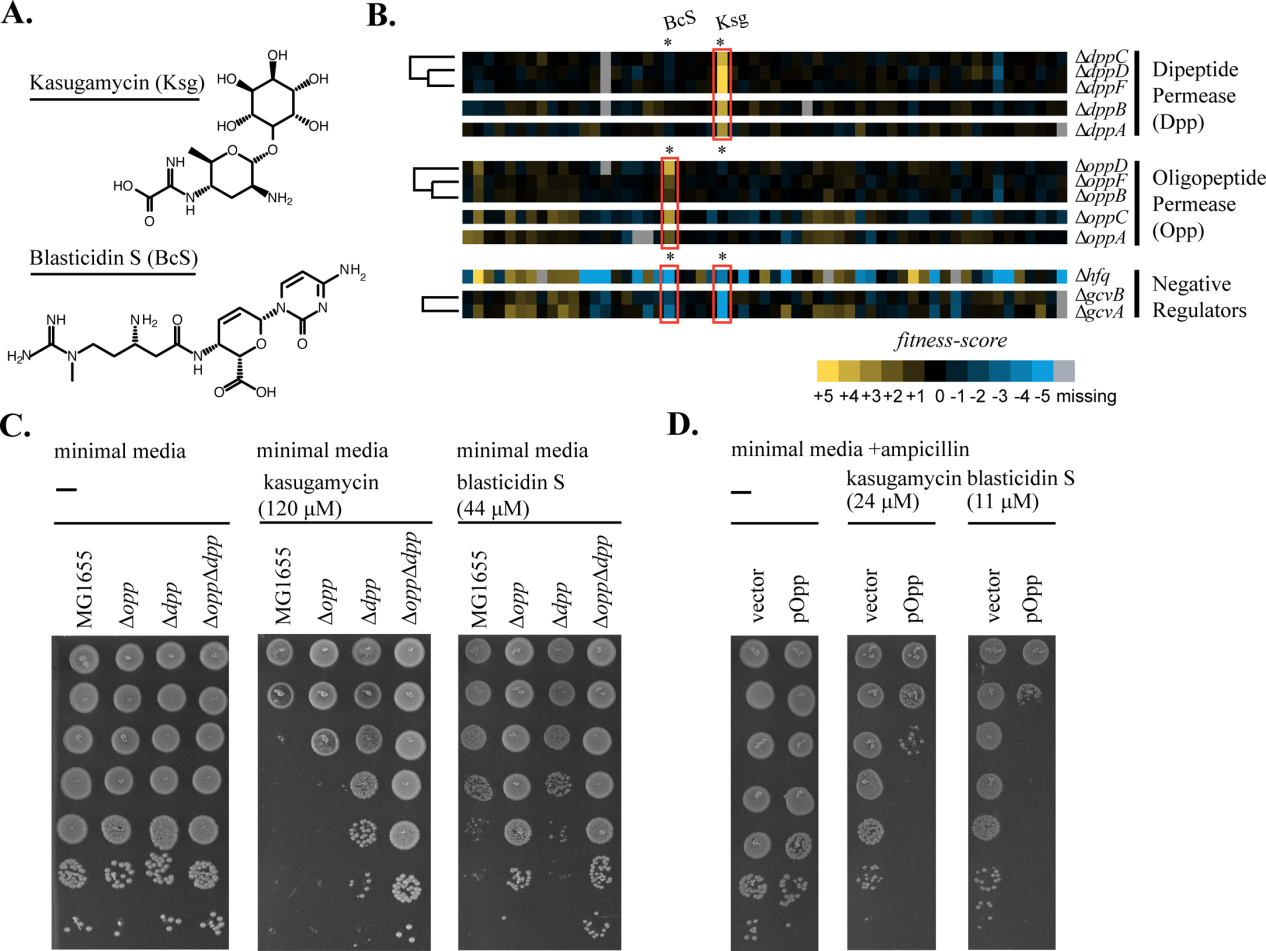
Peptide ABC-importers determine susceptibility to kasugamycin and blasticidin S in *E*. *coli* K-12. (A) Structures of kasugamycin (Ksg) and blasticidin S (BcS). (B) Deletions of peptide importer genes are resistant to kasugamycin and blasticidin S. The heat-map of fitness-scores for dipeptide permease (Δ*dppA*, Δ*dppB*, Δ*dppC*, Δ*dppD*, and Δ*dppF*), oligopeptide permease (Δ*oppA*, Δ*oppB*, Δ*oppC*, Δ*oppD*, and Δ*oppF*), and their negative regulators (Δ*hfq*, Δ*gcvA*, and Δ*gcvB*) for the entire set of new stresses is shown. Ksg and BcS are highlighted within the heatmap. (C) Deletions of each peptide permease operon show an increase in resistance to Ksg and BcS. 10-fold spot dilutions are shown for operon deletions Δ*opp*, Δ*dpp* and the double mutant Δ*opp* Δ*dpp* (D) Overexpression of *opp* results in a decrease in resistance to Ksg and BcS. 10-fold spot dilutions of cells with the high copy vector pDSW204 containing the *opp* operon (pOpp) grown without induction indicate decreased resistance to both Ksg and BcS relative to the empty vector control (vector).

Hierarchical clustering of the fitness scores revealed a potential connection between the translation inhibitors kasugamycin (Ksg), an inositol-based aminoglycoside, blasticidin S (BcS), an aminonucleoside, (Fig 3A) and the major peptide ABC-importers of *E*. *coli* K-12; oligopeptide permease (Opp) and dipeptide permease (Dpp)[33–36] (Fig 3B). Dpp import is specific for dipeptides [37] while Opp can import peptides less than 5 amino acids in length [38].

Cells harboring deletions of each component of the Dpp complex were resistant to Ksg and three clustered (Δ*dppC*, Δ*dppD*, and Δ*dppF*) with Ksg resistance as their major phenotype. Similarly, most deletions of Opp subunits were resistant to BcS and three clustered (Δ*oppB*, Δ*oppD*, and Δ*oppF*). In addition, deletions of the negative regulators of Opp and Dpp expression (Δ*ΔgcvB*, *Δhfq*, and *ΔgcvA*) led to hypersensitivity to both Ksg and BcS.

We investigated these phenotypes further by constructing precise deletions of the ABC-importer operons (Δ*opp* and Δ*dpp*) and analyzing their phenotypes in MG1655, the standard wild-type background, growing in minimal media at a neutral pH. This growth condition enhances drug efficacy [16,17] and is associated with increased expression of *opp* and *dpp* [39,40]. Spot dilution tests revealed that the operon deletion strains grew equivalently to MG1655 in minimal media (Fig 3C, left), but were resistant to Ksg (Fig 3C, middle) and BcS (Fig 3C, right). Individual deletion strains Δ*opp* and Δdpp were resistant to Ksg, but the highest level of Ksg resistance was conferred by the Δ*opp* Δ*dpp* double mutant. This suggested that both complexes participate in Ksg import. For BcS, Δ*opp* alone was sufficient to confer high-level resistance.

Consistent with elevated expression of the importers leading to sensitivity to Ksg and BcS, expression of the entire Opp operon from a plasmid (pOpp) was sufficient to confer sensitivity to both drugs (Fig 3D). Furthermore, the sensitivities of Δ*gcvA* and Δ*gcvB* to Ksg and BcS were completely suppressed in a Δ*opp* Δ*dpp* background (S2 Fig) confirming that these phenotypes were due to overexpression of Opp and Dpp.

### Opp and Dpp directly import kasugamycin and blasticidin S

**Fig 4:**
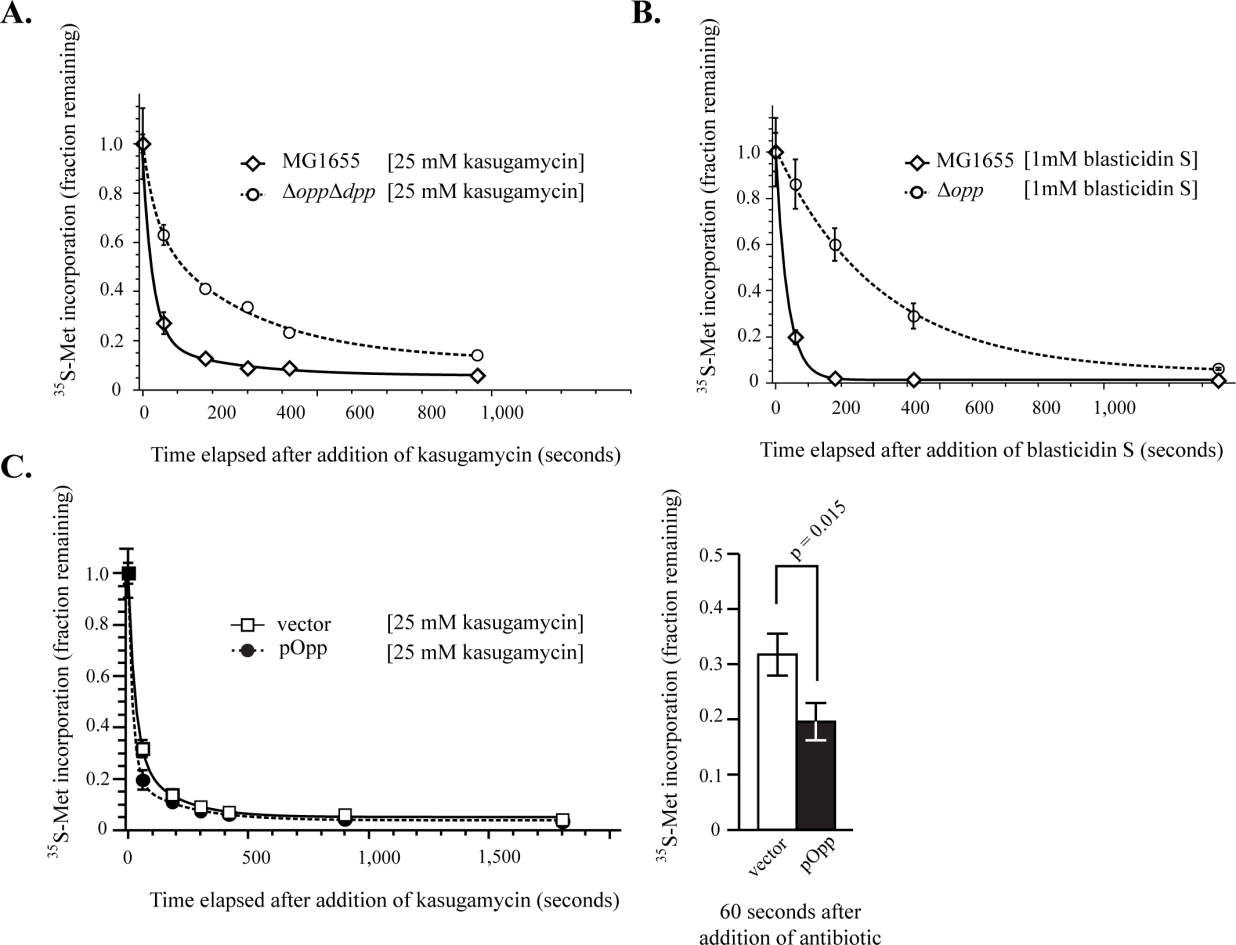
Peptide ABC-importers determine the rate of translation inhibition by kasugamycin and blasticidin S. Altered kinetics of translation inhibition, as measured by ^35^S-methionine incorporation, serve as a proxy for changes in antibiotic uptake rates. (A) Deletion of *opp* and *dpp* slows the rate of translation inhibition following addition of 25mM kasugamycin (B) Deletion of *opp* slows the rate of translation inhibition by 1mM blasticidin S. Error bars represent standard deviation of technical replicates (n = 3) Each experiment was repeated with at least one biological replicate with similar results. (C) Overexpression of *opp* from a high copy vector increases the rate of translation inhibition by kasugamycin. Error bars represent measurements from two biological replicates (left). Significance was tested using a paired two-tailed student’s t-test (n = 2) at one minute after addition of kasugamycin (right). The kinetics of translation inhibition by kasugamycin was best fit with a double exponential decay function, whereas inhibition by blasticidin S was best fit using a single exponential decay function.

Both Ksg and BcS must transit through the inner membrane into the cytoplasm before they can bind to the ribosome and inhibit translation. The rate at which the drug enters the cytoplasm is likely the rate-limiting step for drug action. If so, an *in vivo* assay quantifying the rate translation is inhibited after drug treatment is a reasonable proxy for the rate of drug import. We used the amount of ^35^S-methionine (^35^S-Met) incorporated in a 1 min pulse as a measure of translation rate, and then quantified its rate of decline after addition of antibiotic. We found that the rate of translation inhibition after addition of Ksg (Fig 4A) and BcS (Fig 4B) was dependent on the presence of the peptide ABC-importers, with the ABC-importer deletion strains showing a 7 to 10-fold slower rate of decrease in translation than MG1655. In contrast, the rate of translation inhibition by kanamycin and spectinomycin was similar between MG1655 and the operon deletion strains (S3 Fig). This indicated that, like the antibiotic sensitivities, the effect of deleting these importers on antibiotic uptake was specific to Ksg and Bls. Finally, although less striking in magnitude, the rate of translation inhibition by kasugamycin was significantly faster when the *opp* operon was overexpressed (p<0.02 Paired Student’s t-test) (Fig 4C). These results provided evidence that the observed antibiotic sensitivities were due to altered drug uptake.

**Fig 5:**
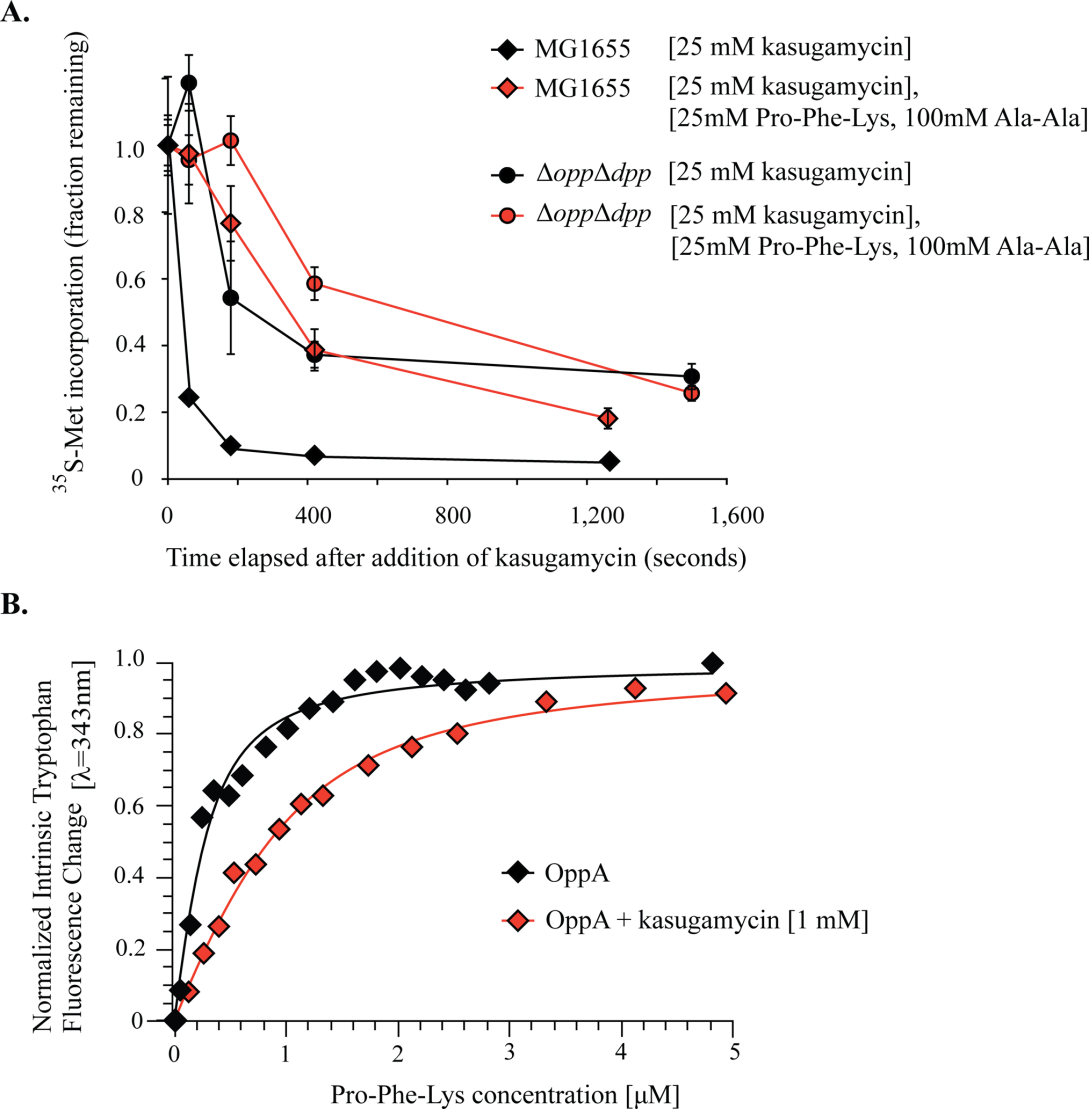
The peptide ABC-importers directly import kasugamycin. (A) The effect of peptide competitors for Dpp (Ala-Ala) and Opp (Pro-Phe-Lys) on the kinetics of inhibition of ^35^S-methionine incorporation. Addition of peptide competitors slowed the rate of translation inhibition by kasugamycin for wild-type *E*. *coli* but had a negligible effect for Δ*opp* Δ*dpp*. Error bars represent standard deviation of technical replicates (n = 3). This experiment was repeated with one biological replicate with similar results. (B) PFK binding to purified OppA induced an increase in intrinsic tryptophan fluorescence, as measured at 343 nm. Addition of 1 mM kasugamycin increased the effective concentration of PFK required to reach half-maximal fluorescence shift. Independent experiments with unique protein purifications had similar results. Equilibrium binding of PFK with and without kasugamycin was fit with a quadratic model that incorporates ligand-depletion.

To further test the hypothesis that Ksg was being directly imported by Opp and Dpp, we used an *in vivo* substrate competition assay (Fig 5A) to test whether the presence of high affinity substrates of Opp (Pro-Phe-Lys; PFK) and Dpp (Ala-Ala; AA) [41,42] competed with Ksg for uptake. Indeed, when Ksg was co-administered with these substrates, the rate of translation inhibition by Ksg was slowed dramatically, approximating that of the Δ*opp* Δ*dpp* double mutant strain. In contrast, the inhibition rate of a Δ*opp* Δ*dpp* strain was virtually insensitive to the addition of competitors.

We next used an *in vitro* binding assay to test for a direct interaction between Ksg and OppA. We expressed and purified His-tagged OppA and tested for Ksg binding using intrinsic tryptophan fluorescence. Addition of 1 mM Ksg markedly increased the apparent K_D_ of OppA for its high-affinity substrate PFK [43,44,42] (Fig 5B) but did not appreciably shift OppA fluorescence when added alone. A similar phenomenon has been shown for DppA, for which the relative change of fluorescence differs markedly even between natural high affinity peptide substrates [41]. While we cannot exclude the possibility that Ksg only indirectly alters OppA affinity for PFK in our *in vitro* assays, the increased apparent K_D_ of OppA for PFK in the presence of Ksg is consistent with competitive binding between Ksg and PFK.

### Opp and Dpp work independently to import Ksg

**Fig 6:**
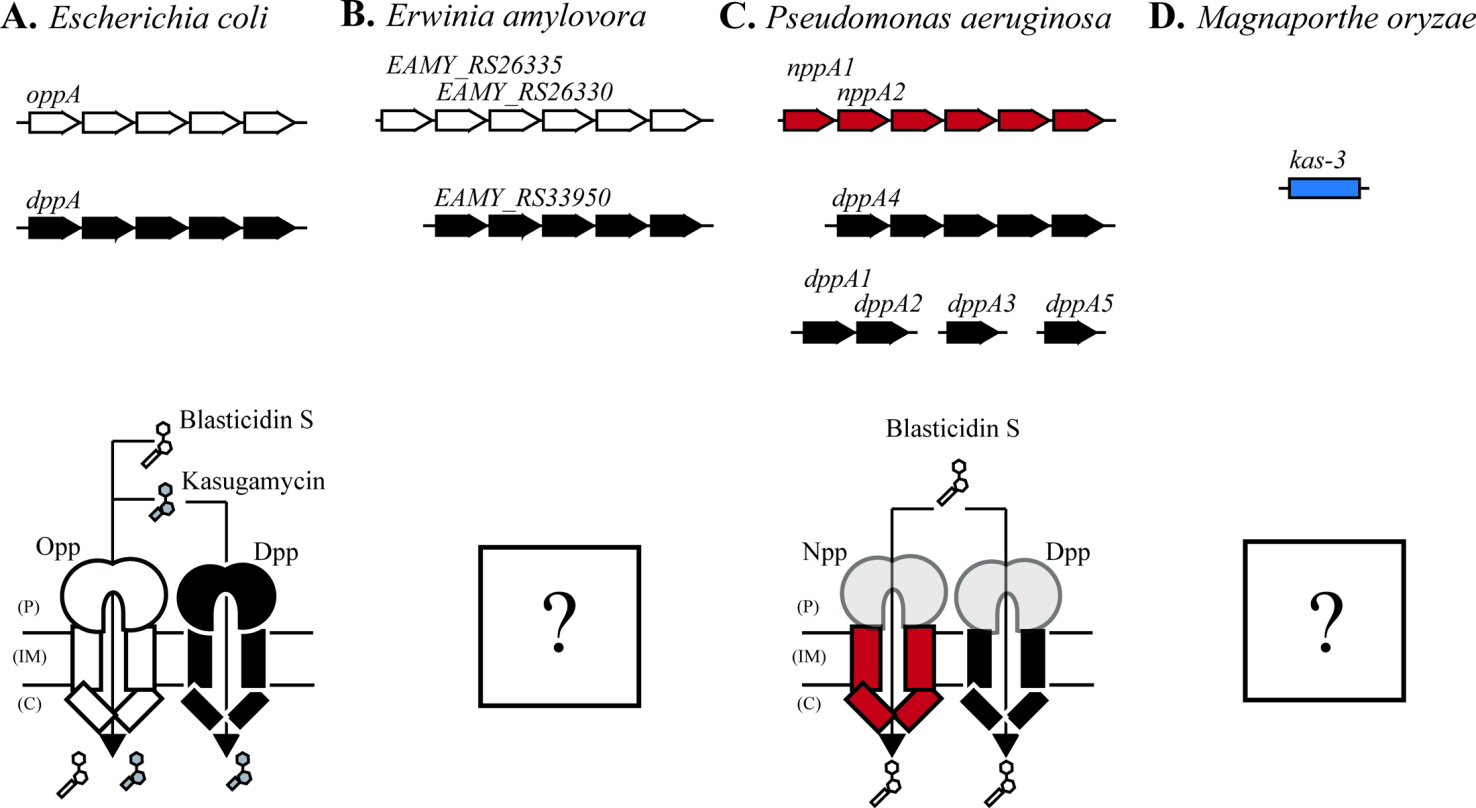
A common import pathway for kasugamycin and blasticidin S is likely to be conserved across multiple species. Genetic context is shown for importer genes likely to be involved in kasugamycin and blasticidin S uptake for multiple species. Sequence homologues of *opp* are shown in white. Sequence homologues of *dpp* are shown in black. The importer *npp* is shown in red. The proposed eukaryotic uptake gene from *Magnaporthe oryzae* is shown in blue. Genes that encode solute-binding proteins are named in each species. Periplasm (P), Inner Membrane (IM), and Cytoplasm (C) are labeled. (A) The *opp* and *dpp* operons in *E*. *coli* contribute to uptake of both kasugamycin and blasticidin S. The reference genome used was MG1655 (U00096.3). (B) *Erwinia amylovora* has high sequence homology to *E*. *coli* for both *opp* and *dpp*, and a duplication of the *oppA* gene. Uptake pathways in this species are unknown but likely to be similar to *E*. *coli*. The reference genome used was CFBP1430 (FN434113.1) (C) *Pseudomonas aeruginosa* has no clear sequence homologues of *opp* and multiple copies of *dppA*. Both the *npp* and *dpp* importers appear to uptake blasticidin S, but the responsible solute binding proteins have not been identified. The reference genome used was UCBPP-PA14 (CP000438.1) (D) *Magnaporthe oryzae* has no sequence homologues of *oppA* or *dppA*, but cross-resistance between kasugamycin and blasticidin S indicate that an analogous importer may be operating in this eukaryote. The gene responsible for cross-resistance was tentatively named *kas-3* but this gene has not been mapped [48]. The reference genome used was 70–15 (AACU00000000.3).

The solute binding protein (SBP) of each peptide ABC-importer freely diffuses in the periplasm, binding its substrate and delivering it to the pore of the complex (Fig 6A). In some cases, a single SBP has been shown to interact with the pores of multiple importers [45–47]. In particular, the SBP MppA has been shown to interact with both the Opp [46] and Dpp [47] pore in *E*. *coli*, depending on its substrate. Furthermore, DppA moonlights as the chemoreceptor for the peptide chemotaxis system of *E*. *coli* [37]. We refer to this phenomenon as crosstalk. To test whether crosstalk between importers or to other systems contributed to the import pathway for Ksg or BcS, we measured genetic interactions between components of the two complexes using the gold-standard assay for drug efficacy: MIC changes determined from a liquid 2-fold dilution series (see Methods).

We first validated that the MIC changes of the peptide importer mutants were consistent with observations from the spot test assay. The single mutant Δ*opp* was sufficient to confer an increase in the MIC for blasticidin S by more than an order of magnitude. For kasugamycin, Δ*opp* conferred a 2-fold MIC increase, and the double mutant Δ*opp* Δ*dpp* exhibiting a a 4-fold MIC (Table 2). *Δdpp* did not confer greater than a two-fold increase in MIC in this assay. Overall, liquid MIC measurements were consistent with the original observation that BcS was imported through Opp while Ksg was imported through both Opp and Dpp.

**Table 2:** Genetic interactions of the peptide ABC-importer subunits.

Strain	MIC Kasugamycin (μg/mL) (Minimal Media)	MIC Blasticidin S (μg/mL) (Minimal Media)
*E*. *coli* K-12 MG1655	50	3–4
Δ*opp*	100	60
Δ*oppA*	100	60
Δ*oppB*	100	60
Δ*opp* Δ*dpp*	200	60
Δ*oppA* Δ*dppA*	200	60
Δ*oppB* Δ*dppB*	200	60

We then tested for crosstalk during illicit transport. We asked whether deleting the SBP had the same quantitative effect on drug efficacy as deleting the pore. If crosstalk occurred, deleting a single SBP would provide less resistance than deleting the pore because all SBPs that deliver that substrate to the same pore must be removed to completely eliminate transport. For Opp, deleting the SBP (Δ*oppA*), the pore (Δ*oppB*), or the entire complex (Δ*opp*) all resulted in the same extent of resistance to both Ksg and BcS (Table 2). This is consistent with OppA delivering Ksg and BcS to the Opp pore without crosstalk. Import of Ksg through Dpp also depended on DppA alone. Deleting both SBPs (Δ*oppA* Δ*dppA*) or both pores (Δ*oppB* Δ*dppB*) conferred the same degree of resistance to Ksg as deleting both operons (Δ*opp* Δ*dpp*), excluding a contribution from any additional unidentified SBPs in Ksg import (Table 2). We suggest a straightforward model in *E*. *coli* K-12 in which Opp directly imports both Ksg and BcS, Dpp directly imports Ksg, and there is no crosstalk for either complex (Fig 6A).

## Discussion

We report a chemical-genomic screen in *E*. *coli* K-12 focused on antibiotics with poorly characterized modes of action and determinants of resistance. We expect that this chemical-genomic dataset will function as a valuable community resource for generating new hypotheses based on these 26 stresses. Integrating the smaller chemical-genomic screen with a larger resource was critical for extracting more information from the new stresses, and this success sets a precedent for continuing to add to the dataset to characterize new antibiotics and find leads for investigating gene function. Characterizing the mechanisms of resistance to new antibiotics will be valuable for future development by identifying those compounds least likely to face already prevalent antibiotic resistance. The ability to integrate smaller-scale screens with larger resources, which we demonstrate here, will facilitate the economical application of chemical-genomics in drug discovery pipelines.

Starting with one feature in this screen, we describe the illicit transport of blasticidin S (BcS) and kasugamycin (Ksg) through the peptide ABC-importers oligopeptide permease (Opp) and dipeptide permease (Dpp). The peptide ABC-importer family is central to the uptake of multiple antibiotics in bacteria [28–32]. The flexible binding mechanisms used to accommodate the varied side chains of different peptides [49–51] could explain the susceptibility of these importers to being hijacked by so many illicit substrates. However, biophysical details of substrate binding by OppA and DppA are currently limited to natural substrates, so the nature of the interaction between these importers and their illicit substrates remains unclear. Purified OppA appears to have a weak affinity for Ksg, but Opp rapidly imports this compound *in vivo* nonetheless. Further characterization of the binding determinants of OppA and DppA for Ksg could be highly informative for defining the minimal requirements of binding and import by these promiscuous complexes.

The shared uptake pathway for Ksg and BcS is likely to be conserved among many of the pathogens against which these antibiotics are regularly used [52–54], although mechanistic details may differ from species to species. Both operon structure and sequence of the SBPs are highly conserved between *E*. *coli* and *E*. *amylovora*, but *E*. *amylovora* carries a duplication of *oppA* (*EAMY_RS26335* and *EAMY_RS26330*) (Fig 6B) which may complicate the uptake mechanism. In *Pseudomonas aeruginosa*, there are five *dppA* homologues (*dppA1-5*) scattered across the genome and no clear homologue of the *opp* system. Despite this divergence, it was recently shown that both Dpp and a “third” peptide ABC-importer, Npp, contribute to BcS sensitivity (Fig 6C). The exact SBPs that participate in BcS uptake in *P*. *aeruginosa* are currently unknown [31]. There are no sequence homologues of either *oppA* or *dppA* in *Magnaporthe oryzae*. However, strains isolated for resistance to either Ksg (B1-100-4 *kas-3*) [48] or BcS (Bu7) [55] can display cross-resistance. Given their distinct chemical structures, unique binding sites on the ribosome [56], and demonstration of a shared uptake mechanism in *E*. *coli*, this cross-resistance is most likely due to a simultaneous loss of uptake of both compounds. Indeed, a functionally equivalent importer that remains to be identified could be responsible for uptake of both Ksg and BcS in this fungal pathogen (Fig 6D).

Cross-resistance from Ksg treated fields [57] suggests that loss of uptake is a common resistance mechanism in *M*. *oryzae*. However, naturally occurring Ksg-resistant ABC-importer mutants have yet to be isolated from any bacterial pathogens. Redundancy between import complexes may reduce the occurrence of this type of mechanism. The fitness impacts of deleting any of the *opp* or *dpp* genes are negligible within the integrated screen, but fitness effects have not been measured in the context of infection and these mutants may be quickly outcompeted in this environment. In addition, there are a number of alternative resistance mechanisms for Ksg including efflux [58], chemical modification of the antibiotics [53], and alteration of the binding site [52,59]. Further research in these organisms will be critical for predicting the contribution that loss of uptake will have on resistance in an agricultural or medical setting. The mechanism we describe is an example of one discovery from our chemical-genomic screen with broad implications for antibiotic resistance in different species and we expect many more discoveries to be made from this large-scale dataset.

## Methods

### Media, growth conditions, strains, plasmids, and oligos

Chemical sensitivity screens used LB Lennox agar plates (1% (w/v) tryptone, 0.5% (w/v) yeast extract, 90 mM sodium chloride, 2% (w/v) bacto agar) unless otherwise specified. M9 minimal plates used in the screen contained M9 salts, 0.2% (w/v) glucose, and 2% (w/v) bacto agar. Media for kasugamycin and blasticidin S sensitivity was M9 minimal supplemented with metal cations and buffered at pH 7.5 (M9 salts, 0.4% (w/v) glucose, 100 μM magnesium sulfate, 100 μM calcium chloride, 5 μM iron(III) chloride, 20 mM Tris-HCl, pH 7.5). To promote high translation rates, media used for ^35^S-methionine incorporation was MOPS EZ rich (-Met), 0.4% (w/v) glucose (Teknova M2101, M2102, M2103, M2109, G0520).

Ordered libraries grown during the chemical-genomics screens were incubated at 37°C until the majority of colonies reached a defined size (~8 hours) then a photograph was taken of the plate. Spot-dilution plates were grown roughly 24 hours at 37°C before a photograph was taken. For MIC determination, cultures were grown in deep 96-well plates for 24 hours in an Infors-HT shaker, 900 r.p.m., 37°C. For ^35^S-methionine incorporation, cultures were grown at 37°C in baffled flasks in a gyrotory® water bath shaker at 350 r.p.m.

Strains used in this study are listed (S2 Table). The KEIO deletion library is derived from BW25113 (F^-^ λ^-^ Δ*(araD-araB)657* Δ*lacZ4787(*::*rrnB-3) rph-1 Δ(rhaD-rhaB)568 hsdR514*) [60]. All experiments subsequent to the chemical-genomics screen were conducted using strains of MG1655 (F^-^ λ^-^ *ilvG*^-^ *rfb-50 rph-1*) [61]. Operon deletions were generated using λ-red recombineering to replace the operon with a kanamycin resistance cassette amplified from pKD4. The resistance cassette was subsequently excised to generate deletions with a single FRT site as a scar. Plasmids and oligonucleotides used in the study are listed (S2 Table).

### Data collection and processing

The chemical genomics screen was conducted using the same methodology as reported previously [8] with few modifications. Ordered libraries were arrayed on rectangular agar plates, grown in the presence of antibiotic or other stress until the colonies reached a defined average size, and then imaged. One condition, 4°C survival, involved growth of colonies on LB plates at 37°C for 6 hours, and transfer of the colony array to 4°C for 5 weeks. Colonies were then pinned onto a fresh plate and surviving cells were allowed to grow for 6 hours at 37°C before being imaged.

Plate photographs were taken with a Canon Powershot G10, using an in-house assembly to control plate illumination. Images were analyzed using Iris to measure the total intensity of pixels within the colony to calculate an opacity metric. Data filtering, normalization, and calculation of the fitness-score was conducted using an in-house analysis pipeline. Code for the analysis is available online (https://github.com/AnthonyShiverMicrobes/fitness_score.git). Steps added to the original analysis pipeline [18] include simultaneous input and analysis of colony size, opacity, and circularity from Iris (read_data.m), manual removal of data based on plate position (to eliminate false positives from minor pinning problems) (filter_data.m), higher-order surface normalization (incorporating a quartic smoothing function that better describes the systematic errors due to pinning effects for *E*. *coli*) (smooth_data.m), power-transformation of the data (to reduce variability of extreme values) (transform_data.m), and variance normalization of the data (to improve reproducibility of measurements between plates) (RC4_scale.m). The raw data and metadata files for this analysis are available online (http://dx.doi.org/10.5061/dryad.f3kc0).

### Clustering, significant phenotypes and correlations, and network analysis

Unreliable measurements were removed from the dataset at multiple points in the analysis and each condition had a different number of measurements (fitness-scores) that passed analysis. Before data integration and clustering were performed, conditions and strains with less than 75% reliable measurements were removed from analysis. Hierarchical clustering was performed using the Cluster 3.0 [62] command line interface, using Pearson’s correlation and average-value linkage. Data was visualized using Java Treeview [63].

To predict reliable phenotypes and pairwise correlations, we used the method described by Nichols et al. [8] to determine the false-discovery rate (FDR), defined as the fraction of positive test results that are expected to be due to type I error, and set a cut-off for the fitness-score that reflected an FDR of 5%. Using this method, 95% of the cutoff values for negative (sensitization) fitness-scores fell in the range (-2.0,-1.2) while 95% of the cutoff values for positive (resistance) fitness-scores fell in the range (+1.2,+2.1).

To identify gene sets enriched for cold-sensitivity at 10°C, we used Gene Set Enrichment Analysis (GSEA) [64,65] of the fitness-scores for 10°C. We grouped genes according to the functional categories of their COG assignments. Input files for this analysis are available online at (http://dx.doi.org/10.5061/dryad.f3kc0).

### MIC determination

A liquid-broth dilution method was used to determine MIC values for the antibiotics. Fresh colonies were picked, resuspended in minimal media, and diluted to a final O.D._450_ of 5x10^-4^. Antibiotics were added in a 1:2 dilution series spanning a 64-fold dilution range. After 24 hours of growth, 150uL of culture was transferred to a 96-well spectrophotometer plate, and the O.D._450_ was measured using a Varioskan spectrophotometer (Thermo electron corporation). After blank subtraction, culture densities were normalized to a no drug control, and the concentration of the first drug dilution step at which the normalized culture density fell below 10% was defined as the MIC of the drug. This quantitative measure corresponded well with a qualitative metric based on pelleting the cells and visually inspecting the size of cell pellet.

### ^35^S-Methionine incorporation

Overnight cultures of relevant strains were grown inoculated into MOPS rich defined methionine dropout media (MOPS RDM-Met) at a starting O.D._450_ of 5x10^-3^ and grown to an O.D._450_ of 0.2. To quantify translation rate, 900 μL of culture was added to 30 μL of labeling mix (10 μCi L-^35^S-methionine, 50 μM cold L-methionine, in MOPS RDM-Met) (Easytag L-^35^S-Methionine, Perkin Elmer Corp.), incubated for 1 min in the water bath, then quenched with 100 μL of 50%(w/v) trichloroacetate (TCA) and stored on ice. A 100 μL aliquot of the quenched reaction mixture was deposited on a glass fiber filter (Merck Millipore Ltd. APFC02500) and washed with 10% (w/v) TCA followed by 95% (v/v) ethanol. ^35^S-methionine incorporation into the TCA-insoluble fraction was quantified on a scintillation counter (Beckman Coulter LS6500 multipurpose scintillation counter). To follow translation inhibition, saturating concentrations of antibiotics were added to growing cultures. Translation rate was quantified in intervals following addition of drug and normalized to a timepoint taken two minutes before drug treatment. Data was fit to a double exponential decay function (kasugamycin treatment) or single exponential decay function (blasticidin S, kanamycin, spectinomycin) using QtiPlot [66].

### OppA purification and binding assays

The *oppA* gene from *E*. *coli* MG1655 was amplified and cloned into pBAD22 [67] using NcoI and HindIII restriction enzymes. *E*. *coli* BW25113 was then transformed with the resulting plasmid, and over-production was induced in an exponentially growing culture at O.D._600_ of 0.6 by addition of 0.05% (w/v) arabinose. The purification, including partial unfolding of the protein to remove bound substrates, as well as the binding assays followed the protocol in Klepsch et al. [42]. The changes made were clearing of the lysate for 1 hour at 140,000 g (Beckman L8-M ultracentrifuge), replacement of guanidinium hydrochloride with urea for partial unfolding of OppA, and adjusting the 4-morpholineethanesulfonic acid (MES) buffer to a pH of 6.7 instead of 6.0 during purification to increase stability. OppA was highly concentrated and free of visible protein contaminations after Ni-IMAC as judged by SDS-PAGE and staining with Coomassie Blue, and was thus used in binding assays after extensive buffer exchange to remove the imidazole (Milipore Amicon Ultra centrifugation filters, 10,000 MWCO). The spectral characteristics of purified OppA were identical to those reported previously. All Fluorescence assays (Fluoromax-3, Jobin Yvon Horiba) were performed in 20 mM MES pH 6.0 and 150 mM sodium chloride, as described [42]. Data for the normalized change in intrinsic fluorescence was fit to a quadratic function that models ligand depletion [68] in QtiPlot [66].

## Supporting Information

S1 Fig Dataset size determines the cutoff for the statistical significance of gene-pair correlations. (A) More conditions increase the statistical significance of gene-pair correlations. The interquartile range of the distribution of gene-pair correlations, the cutoff for significance using a false discovery rate (FDR) of 5%, and the cutoff for a multiple hypothesis corrected p-value of 5% are plotted against number of conditions sampled from the Nichols et al. dataset [8]. Both methods for determining statistical significance are described in Nichols et al. [8]. Conditions were chosen randomly from the dataset in 4 independent samplings at each position, averages are plotted. Both variation and significance cutoffs decrease with an increasing number of conditions. The IQR (0.25) and FDR-based cutoff (0.78) of the current chemical-genomic screen (N = 57) are similar to a dataset of similar size sampled from Nichols et al. [8], indicating that differences in these statistical measures are due to dataset size only. (B) Integration of the current screen with a larger resource increases the number of statistically significant gene-pair correlations. In addition to reducing the variability of gene-pair correlations, integration of the current chemical-genomic screen with the larger dataset from Nichols et al. [8] lowered the cutoff for statistical significance from 0.78 to 0.47, including a larger fraction of the pairwise correlations between genes.
(TIF)

S2 Fig The double deletion Δ*opp* Δ*dpp* is epistatic to both Δ*gcvA* and Δ*gcvB*. Spot tests are shown for 10-fold dilutions of Δ*gcvA* and Δ*gcvB* in either a wild-type (MG1655) or double deletion Δ*opp* Δ*dpp* background. All mutants grew equivalently in rich media (LB, upper panel), but both Δ*gcvA* and Δ*gcvB* are sensitive to blasticidin S (middle panel) and kasugamycin (lower panel). Deletion of the ABC-importers (Δ*opp* Δ*dpp*) reduced sensitivity to both drugs and further removal of either Δ*gcvA* or Δ*gcvB* had no impact on the sensitivity of Δ*opp* Δ*dpp*.
(TIF)

S3 Fig Deletion of the ABC-importers has a negligible effect on translation inhibition rates of antibiotics other than kasugamycin and blasticidin S. Altered kinetics of translation inhibition, as measured by ^35^S-methionine incorporation, serve as a proxy for changes in antibiotic uptake rates. **A)** Deletion of *opp* and *dpp* has a minor effect on the rate of translation inhibition by the streptamine-containing aminoglycoside kanamycin (1mM). **B)** Deletion of opp and dpp has no detectable effect on the relatively fast inhibition kinetics of the aminocyclitol spectinomycin (2mM). Error bars represent standard deviation from technical replicates. The kinetics of translation inhibition for both kanamycin and spectinomycin were best fit using a single exponential decay function.
(TIF)

S1 Text Supporting Information references. (DOCX)

S1 Dataset The integrated fitness-score dataset. A table of fitness scores for genes (columns) by conditions (rows) suitable for clustering [62] and other downstream analyses. Conditions are labelled with the condition name, concentration in square brackets ‘[]’, and “batch” number in curly brackets ‘{}’. The batch groups conditions that were measured in the same experiment and normalized as a group. Conditions from Nichols et al. [8] were assigned batch “0”. Gene names are used to label the mutation. Unless otherwise specified, the mutations are precise gene deletions.
(TXT)

S1 Table Cold-sensitive genes from the screen. (DOCX)

S2 Table Strains and plasmids used in this study. (DOCX)
