# Correction: A Chemical-Genomic Screen of Neglected Antibiotics Reveals Illicit Transport of Kasugamycin and Blasticidin S

**DOI:** 10.1371/journal.pgen.1006902

**Published:** 2017-07-21

**Authors:** Anthony L. Shiver, Hendrik Osadnik, George Kritikos, Bo Li, Nevan Krogan, Athanasios Typas, Carol A. Gross

This Research Article includes chemical genetics data which are shown in Fig 1. Whilst revisiting the work presented in the paper, the authors identified two issues with these data. First, the published dataset is not the final version, as it lacks late-stage changes that were made during the normalization of the colony opacity measurements. Second, five conditions that were run multiple times had the repeat runs reported independently, thus affecting the estimate of pairwise gene correlations. The authors have corrected both of these issues in the dataset and are issuing this correction notice to provide readers with access to the corrected data.

**Fig 1:**
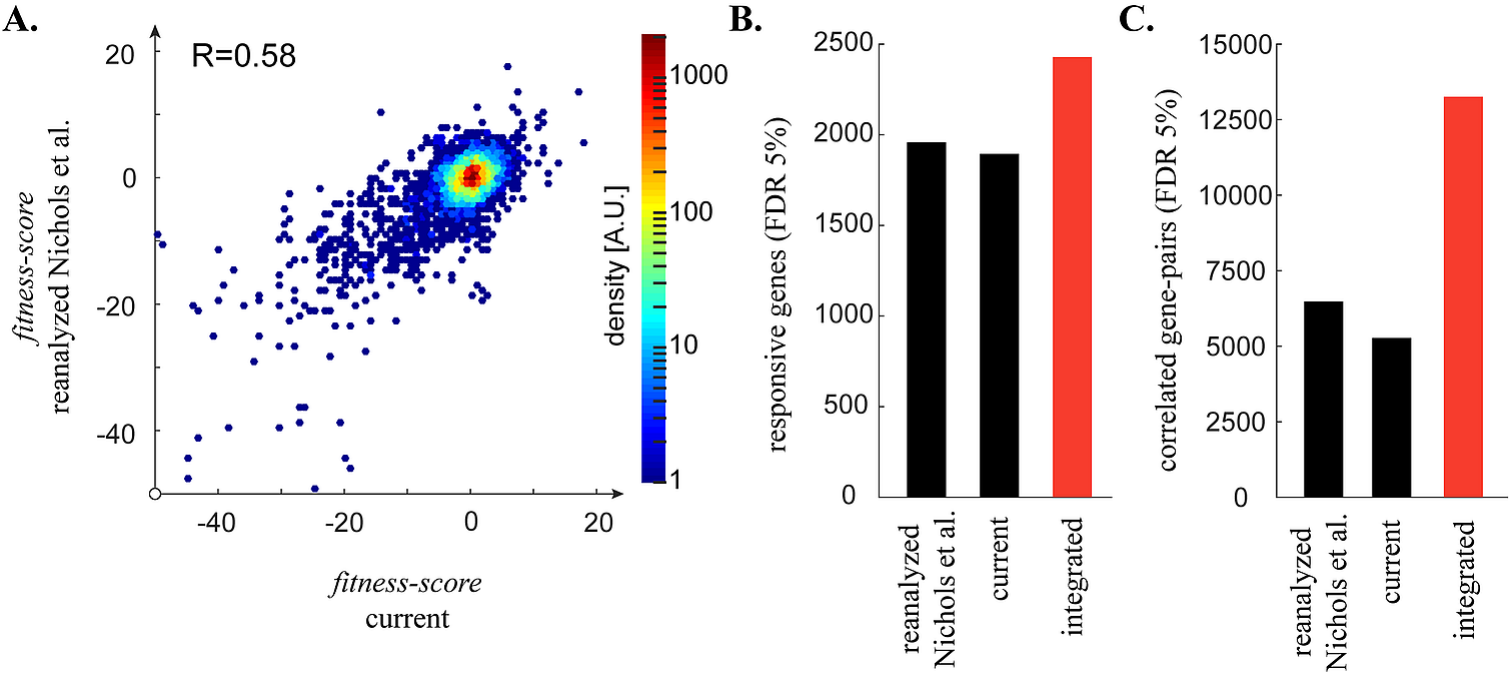
The chemical genomic screen expands our view of gene function and intrinsic resistance in *E*. *coli* K-12. (A) A scatter plot of individual fitness-scores for conditions in both screens (n = 12). Measurements between screens are reproducible, with a Pearson’s correlation of 0.58. B) Conditional-phenotypes were assigned using a stress-specific cutoff for fitness-scores that allowed a false discovery rate (FDR) of 5%. A responsive gene is defined as a gene with at least one phenotype in the dataset. Differences in the number of responsive genes in this dataset as compared to that reported by Nichols et al. predominantly reflects the fact that only 73% of the original conditions were reanalyzed. C) Significant correlations between genes were determined using a cutoff for Pearson’s correlation that allowed an FDR of 5%.

The direct effect of these issues is restricted to the results in Fig 1B and 1C. These changes have a slight effect on fitness scores, number of responsive genes, and number of correlated gene pairs, but the issues identified have no impact on the overall results or conclusions of the paper, and only marginally affect the numerical values presented. Figs 1, 2 and 3 and S1 Fig, S1 Dataset, S1 Table and the legends for Fig 1, S1 Fig and S1 Dataset have been corrected to reflect the new dataset. All other legends, figures and supporting information remain unchanged.

**Fig 2:**
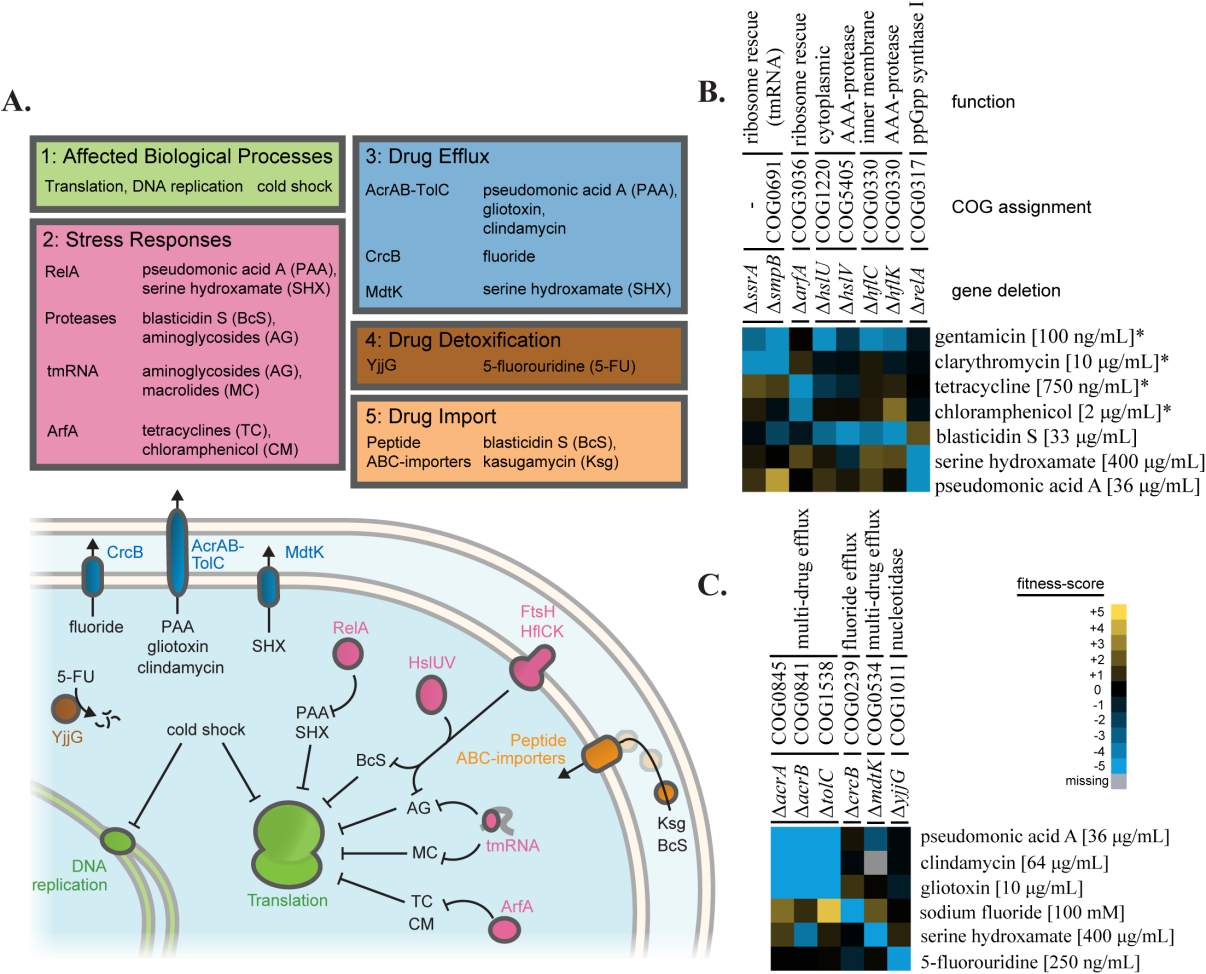
A diverse set of pathways contribute to antibiotic resistance. Fitness-scores from the integrated dataset connected antibiotic resistance to multiple biological pathways. (A) Genes with different resistance mechanisms (target pathway, stress response, drug efflux, detoxification, and drug import) that protect against stresses from the current screen are organized according to mechansim. (B) A heatmap of fitness-scores for translation related genes. Sensitivities of deletions of various translation associated factors distinguish between drug families targeting translation. Sensitivities to aminoglycosides (gentamicin), macrolides (clarithromycin), tetracyclines (tetracycline), chloramphenicol, and tRNA synthetase inhibitors (pseudomonic acid A and serine hydroxamate) are shown. Fitness-scores from the integrated dataset that were determined in Nichols et al. [8] are marked with an asterisk (*). (C) A heatmap of fitness-scores for genes related to drug efflux and detoxification. Multiple drugs from the new screen were connected with the major efflux pump of *E*. *coli*, AcrAB-TolC.

**Fig 3:**
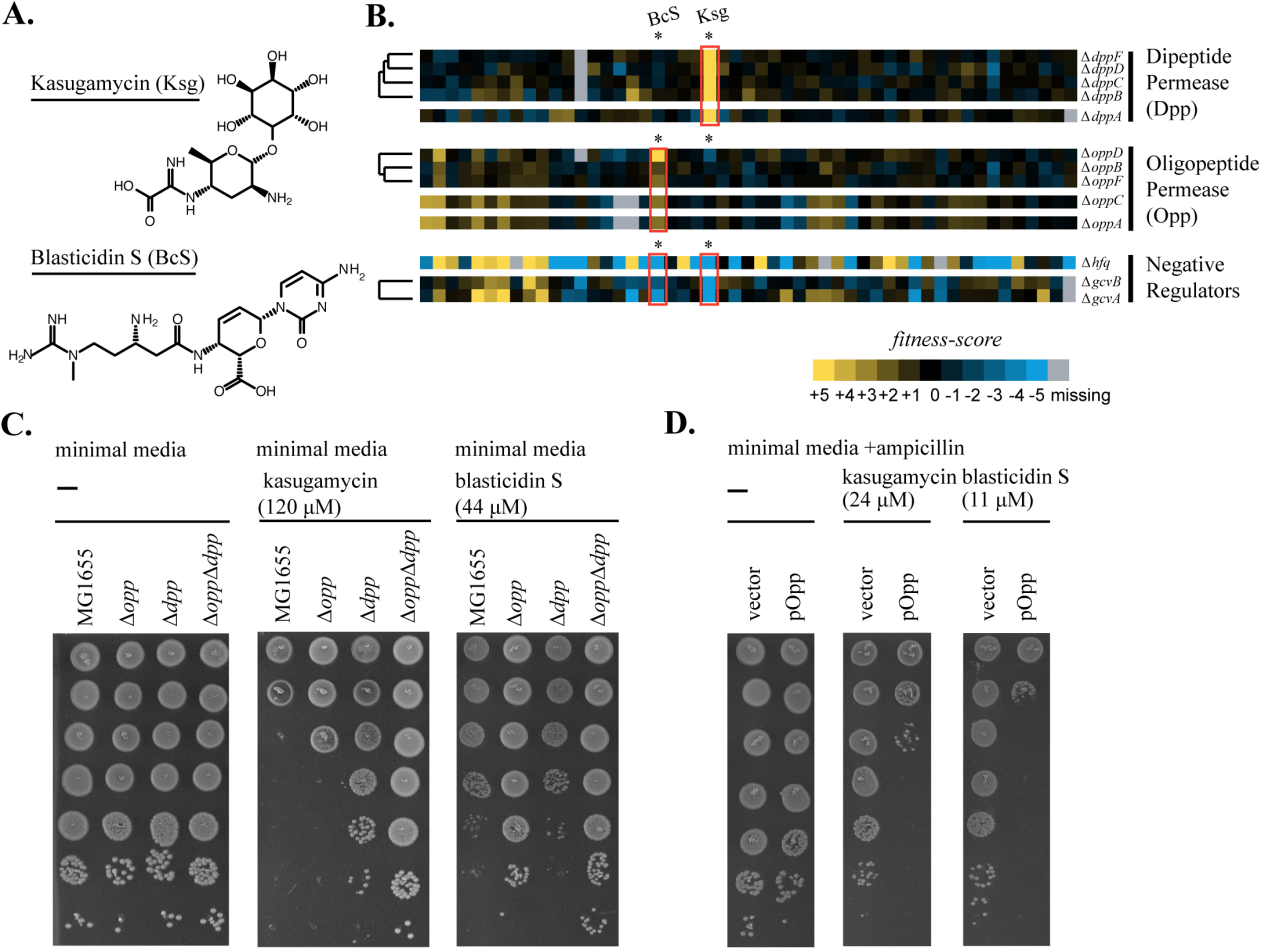
Peptide ABC-importers determine susceptibility to kasugamycin and blasticidin S in *E*. *coli* K-12. (A) Structures of kasugamycin (Ksg) and blasticidin S (BcS). (B) Deletions of peptide importer genes are resistant to kasugamycin and blasticidin S. The heat-map of fitness-scores for dipeptide permease (Δ*dppA*, Δ*dppB*, Δ*dppC*, Δ*dppD*, and Δ*dppF*), oligopeptide permease (Δ*oppA*, Δ*oppB*, Δ*oppC*, Δ*oppD*, and Δ*oppF*), and their negative regulators (Δ*hfq*, Δ*gcvA*, and Δ*gcvB*) for the entire set of new stresses is shown. Ksg and BcS are highlighted within the heatmap. (C) Deletions of each peptide permease operon show an increase in resistance to Ksg and BcS. 10-fold spot dilutions are shown for operon deletions Δ*opp*, Δ*dpp* and the double mutant Δ*opp* Δ*dpp* (D) Overexpression of opp results in a decrease in resistance to Ksg and BcS. 10-fold spot dilutions of cells with the high copy vector pDSW204 containing the *opp* operon (pOpp) grown without induction indicate decreased resistance to both Ksg and BcS relative to the empty vector control (vector).

These changes also affect several sentences in the Results section and the correct sentences are provided below.

- Sentence one of the first paragraph under the sub-heading “The chemical-genomic screen substantially expands known connections in *E*. *coli*”:
  We tested the sensitivities of 3975 mutants of *E*. *coli* K-12 to 51 stresses, split between new and previously screened conditions.
- Sentences four and five of the second paragraph under the subheading “The chemical-genomic screen substantially expands known connections in *E*. *coli*”:
  We identified more than 5,000 conditional-phenotypes for the 26 new stresses, as well as more than 450 additional responsive genes from the 51 stresses tested (13% of the library) (Fig 1B). The conditional-phenotypes that identified additional responsive genes were spread evenly throughout the current screen, ranging from 6 to 54 conditional phenotypes per condition from within the set of new responsive genes.
- Sentence one of the second paragraph under the subheading “Two peptide ABC-importers determine the susceptibility of E. coli K-12 to kasugamycin and blasticidin S”:
  Cells harboring deletions of each component of the Dpp complex were resistant to Ksg and four clustered (Δ*dppB*, Δ*dppC*, Δ*dppD*, and Δ*dppF*) with Ksg resistance as their major phenotype.

## Supporting information

S1 Fig Dataset size determines the cutoff for the statistical significance of gene-pair correlations. (A) More conditions increase the statistical significance of gene-pair correlations. The interquartile range of the distribution of gene-pair correlations, the cutoff for significance using a false discovery rate (FDR) of 5%, and the cutoff for a multiple hypothesis corrected p- value of 5% are plotted against number of conditions sampled from the Nichols et al. dataset [8]. Both methods for determining statistical significance are described in Nichols et al. [8]. Conditions were chosen randomly from the dataset in 4 independent samplings at each position, averages are plotted. Both variation and significance cutoffs decrease with an increasing number of conditions. The IQR (0.25) and FDR-based cutoff (0.76) of the current chemical-genomic screen (N = 51) are similar to a dataset of similar size sampled from Nichols et al. [8], indicating that differences in these statistical measures are due to dataset size only. (B) Integration of the current screen with a larger resource increases the number of statistically significant gene-pair correlations. In addition to reducing the variability of gene-pair correlations, integration of the current chemical-genomic screen with the larger dataset from Nichols et al. [8] lowered the cutoff for statistical significance from 0.76 to 0.46, including a larger fraction of the pairwise correlations between genes.
(TIF)

S1 Dataset The integrated fitness-score dataset. A table of fitness scores for genes (columns) by conditions (rows) suitable for clustering [62] and other downstream analyses. Conditions are labeled with the condition name, concentration in square brackets ‘[]’, and “batch” number in curly brackets ‘{}’. The batch groups conditions that were measured in the same experiment and normalized as a group. Conditions from Nichols et al. [8] were assigned batch “0”. ECK number and gene names are used to label the mutation. Unless otherwise specified, the mutations are precise gene deletions.
(TXT)

S1 Table Cold-sensitive genes from the screen. (DOCX)
