# Supplementary figures and images for: A Chemical-Genomic Screen of Neglected Antibiotics Reveals Illicit Transport of Kasugamycin and Blasticidin S
Source: PLoS Genet. 2016 Jun 29;12(6):e1006124. doi: 10.1371/journal.pgen.1006124 (PMC4927156; doi:10.1371/journal.pgen.1006124)

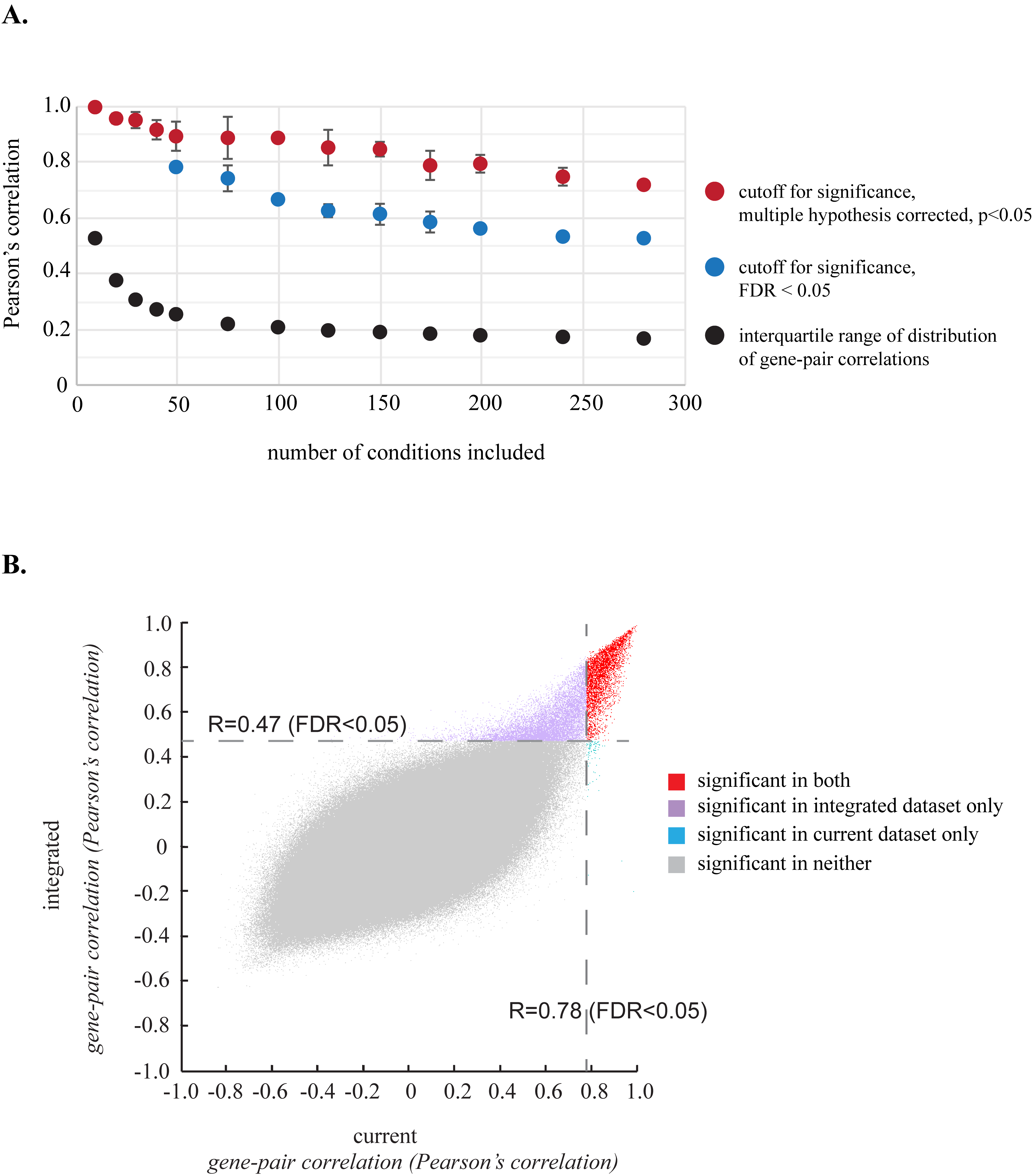

Supplement: S1 Fig — (A) More conditions increase the statistical significance of gene-pair correlations. The interquartile range of the distribution of gene-pair correlations, the cutoff for significance using a false discovery rate (FDR) of 5%, and the cutoff for a multiple hypothesis corrected p-value of 5% are plotted against number of conditions sampled from the Nichols et al. dataset [8]. Both methods for determining statistical significance are described in Nichols et al. [8]. Conditions were chosen randomly from the dataset in 4 independent samplings at each position, averages are plotted. Both variation and significance cutoffs decrease with an increasing number of conditions. The IQR (0.25) and FDR-based cutoff (0.78) of the current chemical-genomic screen (N = 57) are similar to a dataset of similar size sampled from Nichols et al. [8], indicating that differences in these statistical measures are due to dataset size only. (B) Integration of the current screen with a larger resource increases the number of statistically significant gene-pair correlations. In addition to reducing the variability of gene-pair correlations, integration of the current chemical-genomic screen with the larger dataset from Nichols et al. [8] lowered the cutoff for statistical significance from 0.78 to 0.47, including a larger fraction of the pairwise correlations between genes. (TIF) [file pgen.1006124.s001.tif]

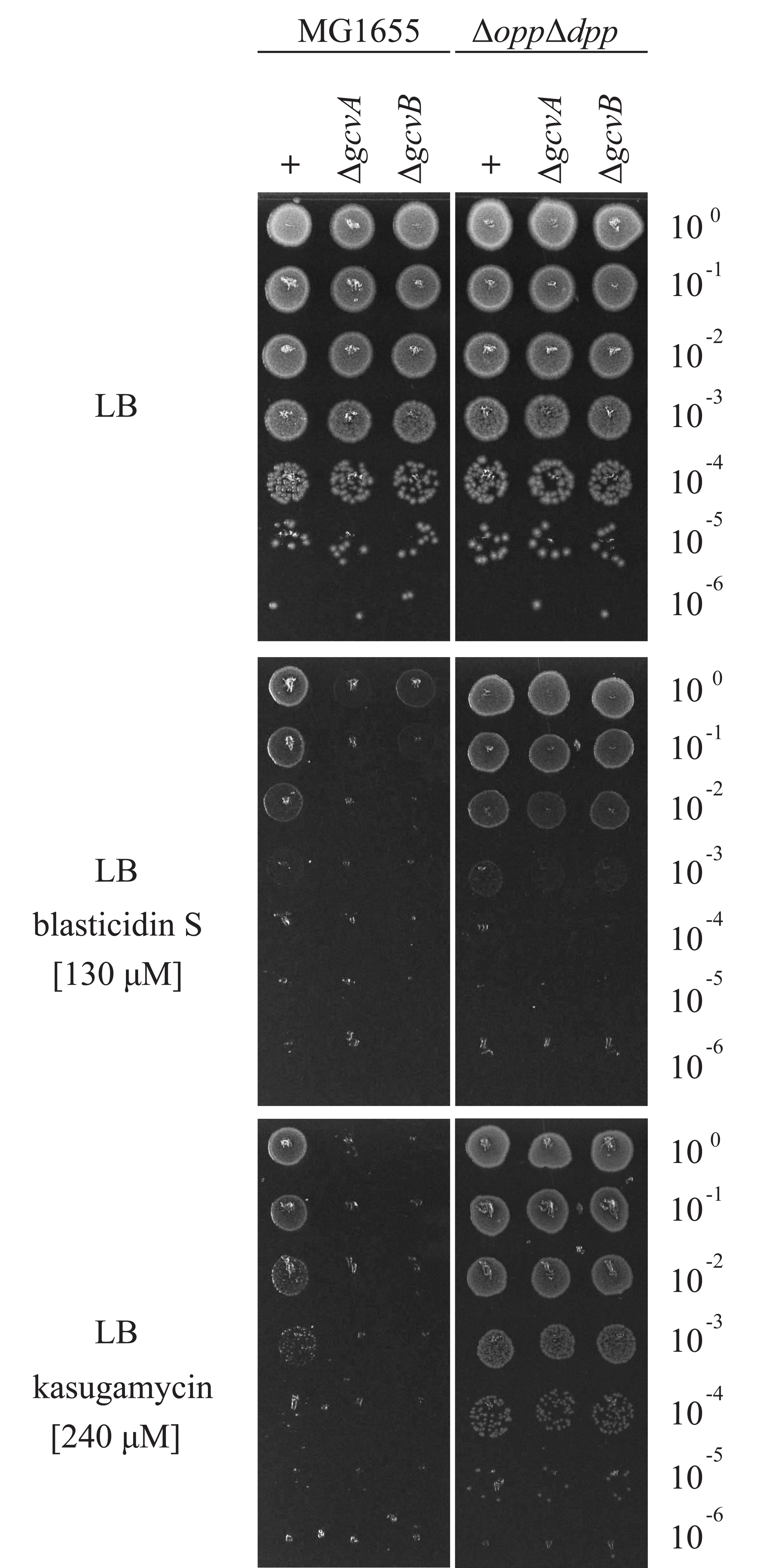

Supplement: S2 Fig — Spot tests are shown for 10-fold dilutions of ΔgcvA and ΔgcvB in either a wild-type (MG1655) or double deletion Δopp Δdpp background. All mutants grew equivalently in rich media (LB, upper panel), but both ΔgcvA and ΔgcvB are sensitive to blasticidin S (middle panel) and kasugamycin (lower panel). Deletion of the ABC-importers (Δopp Δdpp) reduced sensitivity to both drugs and further removal of either ΔgcvA or ΔgcvB had no impact on the sensitivity of Δopp Δdpp. (TIF) [file pgen.1006124.s002.tif]

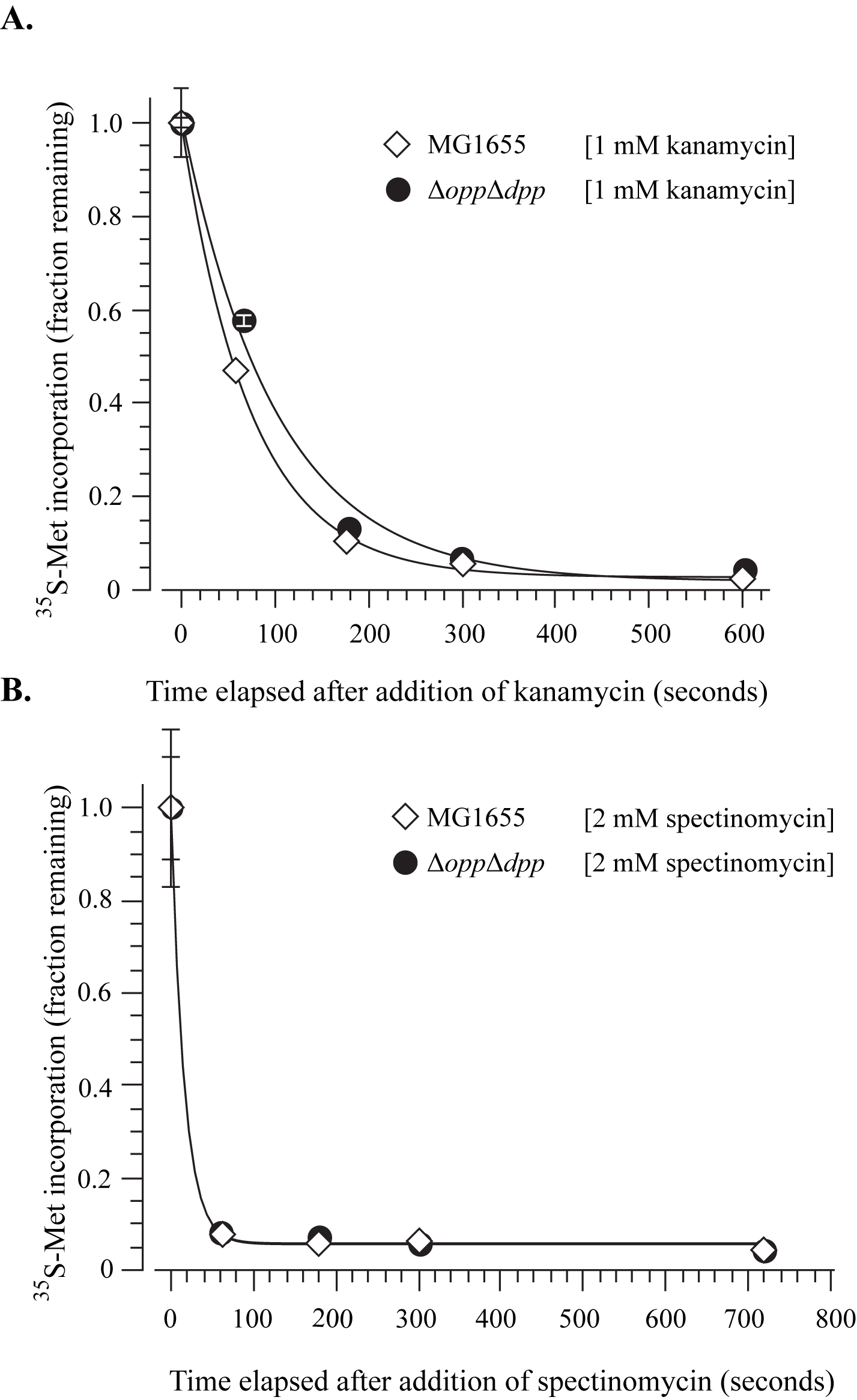

Supplement: S3 Fig — Altered kinetics of translation inhibition, as measured by 35S-methionine incorporation, serve as a proxy for changes in antibiotic uptake rates. A) Deletion of opp and dpp has a minor effect on the rate of translation inhibition by the streptamine-containing aminoglycoside kanamycin (1mM). B) Deletion of opp and dpp has no detectable effect on the relatively fast inhibition kinetics of the aminocyclitol spectinomycin (2mM). Error bars represent standard deviation from technical replicates. The kinetics of translation inhibition for both kanamycin and spectinomycin were best fit using a single exponential decay function. (TIF) [file pgen.1006124.s003.tif]
