# Supplementary material for: A Chemical-Genomic Screen of Neglected Antibiotics Reveals Illicit Transport of Kasugamycin and Blasticidin S
Source: PLoS Genet. 2016 Jun 29;12(6):e1006124. doi: 10.1371/journal.pgen.1006124 (PMC4927156; doi:10.1371/journal.pgen.1006124)
Supplement: S1 Text — (DOCX) [file pgen.1006124.s004.docx]

**Supporting Information References**

69. Choudhury P, Flower AM. Efficient Assembly of Ribosomes Is Inhibited by Deletion of bipA in Escherichia coli. J Bacteriol. 2015 May 15;197(10):1819–27.

70. Polissi A, De Laurentis W, Zangrossi S, Briani F, Longhi V, Pesole G, et al. Changes in Escherichia coli transcriptome during acclimatization at low temperature. Res Microbiol. 2003 Oct;154(8):573–80.

71. Dammel CS, Noller HF. Suppression of a cold-sensitive mutation in 16S rRNA by overexpression of a novel ribosome-binding factor, RbfA. Genes Dev. 1995;9(5):626–637.

72. Charollais J, Dreyfus M, Iost I. CsdA, a cold-shock RNA helicase from Escherichia coli, is involved in the biogenesis of 50S ribosomal subunit. Nucleic Acids Res. 2004;32(9):2751–9.

73. Jiang M, Datta K, Walker A, Strahler J, Bagamasbad P, Andrews PC, et al. The Escherichia coli GTPase CgtAE Is Involved in Late Steps of Large Ribosome Assembly. J Bacteriol. 2006 Oct;188(19):6757–70.

74. Hwang J, Inouye M. The tandem GTPase, Der, is essential for the biogenesis of 50S ribosomal subunits in Escherichia coli. Mol Microbiol. 2006 Sep 1;61(6):1660–72.

75. Britton RA, Lupski JR. Isolation and characterization of suppressors of two Escherichia coli dnaG Mutations, dnaG2903 and parB. Genetics. 1997 Apr;145(4):867–75.

76. Connolly K, Rife JP, Culver G. Mechanistic insight into the ribosome biogenesis functions of the ancient protein KsgA. Mol Microbiol. 2008 Dec;70(5):1062–75.

77. Massé E, Drolet M. R-loop-dependent hypernegative supercoiling in Escherichia coli topA mutants preferentially occurs at low temperatures and correlates with growth inhibition. J Mol Biol. 1999 Nov 26;294(2):321–32.

78. Raychaudhuri S, Conrad J, Hall BG, Ofengand J. A pseudouridine synthase required for the formation of two universally conserved pseudouridines in ribosomal RNA is essential for normal growth of Escherichia coli. RNA. 1998 Nov;4(11):1407–17.

79. Kimura S, Suzuki T. Fine-tuning of the ribosomal decoding center by conserved methyl-modifications in the Escherichia coli 16S rRNA. Nucleic Acids Res. 2010 Mar;38(4):1341–52.

80. Gu XR, Gustafsson C, Ku J, Yu M, Santi DV. Identification of the 16S rRNA m5C967 methyltransferase from Escherichia coli. Biochemistry. 1999 Mar 30;38(13):4053–7.

81. Weiss DS, Chen JC, Ghigo JM, Boyd D, Beckwith J. Localization of FtsI (PBP3) to the septal ring requires its membrane anchor, the Z ring, FtsA, FtsQ, and FtsL. J Bacteriol. 1999 Jan;181(2):508–20.

82. Datsenko KA, Wanner BL. One-step inactivation of chromosomal genes in Escherichia coli K-12 using PCR products. Proc Natl Acad Sci. 2000 Jun 6;97(12):6640–5.
