# Supplementary material for: A Chemical-Genomic Screen of Neglected Antibiotics Reveals Illicit Transport of Kasugamycin and Blasticidin S
Source: PLoS Genet. 2016 Jun 29;12(6):e1006124. doi: 10.1371/journal.pgen.1006124 (PMC4927156; doi:10.1371/journal.pgen.1006124)
Supplement: S1 Table — (DOCX) [file pgen.1006124.s006.docx]

**S1 Table. Cold-sensitive genes from the screen.**

| gene | COG category | Cold shock or translation reference | 10˚C sensitive | 10˚C fitness-score | 16˚C sensitive | 16˚C score |
| --- | --- | --- | --- | --- | --- | --- |
| istR-1 |  |  | TRUE | -11.9 | TRUE | -4.4 |
| typA | T | [69] | TRUE | -10.5 | TRUE | -3.8 |
| **ihfB** | **L** |  | TRUE | -8.7 | FALSE | -0.4 |
| **ihfA** | **L** |  | TRUE | -8.7 | FALSE | -1.0 |
| dinJ | V |  | TRUE | -8.3 | TRUE | -2.0 |
| ydfV |  |  | TRUE | -7.5 | FALSE | -0.5 |
| ymcE |  | [70] | TRUE | -7.4 | FALSE | -1.3 |
| **fusA-SPA** | **J** |  | TRUE | -7.1 | FALSE | -1.2 |
| **rbfA** | **J** | [71] | TRUE | -6.9 | FALSE | 2.0 |
| **deaD** | **L** | [72] | TRUE | -6.7 | TRUE | -4.7 |
| **dnaB-SPA** | **L** |  | TRUE | -6.7 | TRUE | -2.6 |
| smpB | O |  | TRUE | -6.5 | FALSE | -1.5 |
| dcrB |  |  | TRUE | -6.3 | FALSE | -0.6 |
| **dnaA-SPA** | **L** |  | TRUE | -6.3 | TRUE | -4.3 |
| yciM | M |  | TRUE | -6.2 | FALSE | -0.7 |
| hfq | T |  | TRUE | -5.7 | FALSE | -0.3 |
| **pheT-SPA** | **J** |  | TRUE | -5.2 | FALSE | 0.7 |
| crr | G |  | TRUE | -4.8 | FALSE | -1.0 |
| **yjgA** | **J** | [73] | TRUE | -4.7 | TRUE | -2.9 |
| spr | M |  | TRUE | -4.4 | FALSE | -0.1 |
| dnaJ | O |  | TRUE | -4.4 | TRUE | -1.7 |
| ptsN | GT |  | TRUE | -4.3 | TRUE | -1.9 |
| lipB | H |  | TRUE | -4.3 | FALSE | 1.9 |
| **frr-SPA** | **J** |  | TRUE | -4.2 | FALSE | 0.4 |
| glmS-SPA | M |  | TRUE | -4.1 | FALSE | -1.0 |
| ycbK | S |  | TRUE | -4.1 | FALSE | 0.0 |
| nfuA | O |  | TRUE | -4.0 | FALSE | -1.1 |
| der-SPA | R | [74] | TRUE | -3.9 | TRUE | -2.9 |
| rpsF |  | [75] | TRUE | -3.8 | FALSE | -0.9 |
| **rsmA** | **J** | [76] | TRUE | -3.7 | TRUE | -2.9 |
| sdhA | C |  | TRUE | -3.7 | FALSE | 0.1 |
| **rnhA** | **L** | [77] | TRUE | -3.7 | FALSE | -0.6 |
| **prfC** | **J** |  | TRUE | -3.6 | TRUE | -3.6 |
| **rluD** | **J** | [78] | TRUE | -3.5 | TRUE | -1.6 |
| aroK | E |  | TRUE | -3.5 | FALSE | -0.9 |
| gor | C |  | TRUE | -3.5 | FALSE | -0.6 |
| clpX | O |  | TRUE | -3.5 | FALSE | 2.7 |
| **rsmH** | **J** | [79] | TRUE | -3.5 | FALSE | 0.1 |
| ahpC | V |  | TRUE | -3.4 | FALSE | -1.5 |
| **dksA** | **J** |  | TRUE | -3.4 | FALSE | -0.2 |
| yjgZ |  |  | TRUE | -3.3 | FALSE | 1.0 |
| malT | K |  | TRUE | -3.3 | FALSE | 0.0 |
| **rsmB** | **JK** | [80] | TRUE | -3.3 | FALSE | 0.4 |
| ppk | P |  | TRUE | -3.3 | TRUE | -1.8 |
| cbrC | S |  | TRUE | -3.3 | FALSE | 0.5 |
| envC |  |  | TRUE | -3.2 | FALSE | 0.9 |
| wcaJ | M |  | TRUE | -3.1 | FALSE | 0.6 |
| yoaE | PR |  | TRUE | -3.1 | FALSE | 0.2 |
| fliQ | N |  | TRUE | -3.1 | FALSE | -0.2 |
| surA | O |  | TRUE | -3.1 | FALSE | 0.3 |
| gapA-SPA | G |  | TRUE | -3.0 | FALSE | -1.1 |
| yfdG | I |  | TRUE | -2.9 | TRUE | -2.2 |
| hipA | T |  | TRUE | -2.9 | FALSE | -0.3 |
| bamA{dup(218-219)} | M |  | TRUE | -2.8 | FALSE | 1.0 |
| kgtP | GEPR |  | TRUE | -2.8 | FALSE | -0.9 |
| fadR | K |  | TRUE | -2.8 | TRUE | -1.9 |
| **rpmE** | **J** |  | TRUE | -2.8 | TRUE | -2.4 |
| fliF | NU |  | TRUE | -2.8 | FALSE | 0.0 |
| mukE-SPA |  |  | TRUE | -2.8 | FALSE | 1.3 |
| waaF | M |  | TRUE | -2.7 | FALSE | 1.6 |
| grcA | H |  | TRUE | -2.7 | FALSE | 0.1 |
| cydB | C |  | TRUE | -2.7 | FALSE | 0.5 |
| **ybeY** | **J** |  | TRUE | -2.7 | FALSE | 1.0 |
| **dam** | **L** |  | TRUE | -2.7 | FALSE | 1.1 |
| **rpmG** | **J** |  | TRUE | -2.7 | FALSE | -1.4 |
| yhbJ | T |  | TRUE | -2.7 | FALSE | -0.4 |
| murD-SPA | M |  | TRUE | -2.7 | FALSE | -0.7 |
| ydfN |  |  | TRUE | -2.7 | FALSE | 0.2 |
| yaaI |  |  | TRUE | -2.6 | FALSE | -0.2 |
| yqeB | OR |  | TRUE | -2.6 | FALSE | 0.0 |
| cpxA | T |  | TRUE | -2.6 | FALSE | -1.3 |
| gpmM | G |  | TRUE | -2.6 | FALSE | 1.0 |
| dedD | D |  | TRUE | -2.6 | TRUE | -2.0 |
| yneJ | K |  | TRUE | -2.5 | FALSE | 0.7 |
| yejK | S |  | TRUE | -2.5 | FALSE | -0.2 |
| tolR | U |  | TRUE | -2.5 | FALSE | -0.2 |
| glgB | G |  | TRUE | -2.5 | FALSE | -1.4 |
| yggX | PO |  | TRUE | -2.5 | FALSE | -0.7 |
| pstS | P |  | TRUE | -2.5 | FALSE | 0.9 |
| pta | CR |  | TRUE | -2.5 | FALSE | -0.7 |
| murE-C | M |  | TRUE | -2.5 | FALSE | -0.3 |
| fbp | G |  | TRUE | -2.5 | FALSE | 2.4 |
| purR | K |  | TRUE | -2.5 | FALSE | -0.5 |
| rffM | M |  | TRUE | -2.5 | FALSE | -1.1 |
| apaH | T |  | TRUE | -2.4 | FALSE | -0.2 |
| ycjX | R |  | TRUE | -2.4 | FALSE | -0.5 |
| sdhB | C |  | TRUE | -2.4 | FALSE | 2.4 |
| rpiR | K |  | TRUE | -2.4 | FALSE | -0.8 |
| **seqA** | **L** |  | TRUE | -2.4 | FALSE | -0.3 |
| racC |  |  | TRUE | -2.4 | FALSE | -0.3 |
| gmm | F |  | TRUE | -2.4 | TRUE | -1.7 |
| yfiO* | M |  | TRUE | -2.4 | TRUE | -3.2 |
| pmbA | R |  | TRUE | -2.4 | FALSE | -0.8 |
| speB | E |  | TRUE | -2.4 | TRUE | -2.5 |
| rfe | M |  | TRUE | -2.3 | FALSE | -1.0 |
| ydfO | S |  | TRUE | -2.3 | FALSE | 0.2 |
| yejF | Q |  | TRUE | -2.3 | FALSE | -0.1 |
| rffE | M |  | TRUE | -2.3 | FALSE | 0.0 |
| rpoS | K |  | TRUE | -2.3 | FALSE | -0.8 |
| yhcO | K |  | TRUE | -2.3 | FALSE | -0.3 |
| ybjX | S |  | TRUE | -2.3 | FALSE | 0.6 |
| trxA | O |  | TRUE | -2.2 | FALSE | 1.1 |
| pstB | P |  | TRUE | -2.2 | FALSE | -0.2 |
| sixA | T |  | TRUE | -2.2 | FALSE | -0.3 |
| yfcP | N |  | TRUE | -2.2 | FALSE | -0.1 |
| rcsD | T |  | TRUE | -2.2 | FALSE | -0.5 |
| oppA | E |  | TRUE | -2.2 | FALSE | 0.2 |
| secB | U |  | FALSE | NaN | TRUE | -5.1 |
| yfcA | S |  | FALSE | NaN | TRUE | -3.1 |
| cpsG | G |  | FALSE | -0.1 | TRUE | -2.6 |
| fis | K |  | FALSE | 1.6 | TRUE | -2.5 |
| rnlB |  |  | FALSE | -0.9 | TRUE | -2.5 |
| yqjF | S |  | FALSE | 0.4 | TRUE | -2.5 |
| **rpsE-SPA** | **J** |  | FALSE | 0.0 | TRUE | -2.4 |
| yaiS | G |  | FALSE | -0.4 | TRUE | -2.2 |
| isrB |  |  | FALSE | -0.3 | TRUE | -2.1 |
| **infC-SPA** | **J** |  | FALSE | -1.6 | TRUE | -2.1 |
| dmsB | C |  | FALSE | 0.7 | TRUE | -1.9 |
| uup | R |  | FALSE | -1.3 | TRUE | -1.9 |
| **rbn** | **J** |  | FALSE | 0.8 | TRUE | -1.9 |
| ymiA |  |  | FALSE | 0.4 | TRUE | -1.9 |
| flhE |  |  | FALSE | -0.4 | TRUE | -1.9 |
| yegK |  |  | FALSE | NaN | TRUE | -1.8 |
| sbp | P |  | FALSE | 1.3 | TRUE | -1.8 |
| malS | G |  | FALSE | 0.3 | TRUE | -1.8 |
| gabT | E |  | FALSE | 1.0 | TRUE | -1.8 |
| rpoD-SPA | K |  | FALSE | 0.8 | TRUE | -1.8 |
| sgrT |  |  | FALSE | -1.3 | TRUE | -1.8 |
| fpr | C |  | FALSE | -0.6 | TRUE | -1.8 |
| yjfY |  |  | FALSE | -0.1 | TRUE | -1.8 |
| yehD | N |  | FALSE | 0.0 | TRUE | -1.8 |
| ycbC | R |  | FALSE | NaN | TRUE | -1.7 |
| fabF | IQ |  | FALSE | -1.6 | TRUE | -1.7 |
| **polB** | **L** |  | FALSE | -0.8 | TRUE | -1.7 |
| galE | M |  | FALSE | -0.8 | TRUE | -1.7 |
| ndk | F |  | FALSE | 0.2 | TRUE | -1.7 |
| hokD |  |  | FALSE | -0.9 | TRUE | -1.7 |
| sgcB | G |  | FALSE | 0.5 | TRUE | -1.7 |
| acpP-SPA | IQ |  | FALSE | 0.1 | TRUE | -1.6 |
| speE | E |  | FALSE | 0.7 | TRUE | -1.6 |
| ascG |  |  | FALSE | NaN | TRUE | -1.6 |
| ybhU |  |  | FALSE | -0.8 | TRUE | -1.6 |
| appY | K |  | FALSE | -1.5 | TRUE | -1.6 |
| **srmB** | **L** |  | FALSE | -0.9 | TRUE | -1.6 |
| hybE |  |  | FALSE | -0.5 | TRUE | -1.6 |

­
