# Supplementary material for: A Chemical-Genomic Screen of Neglected Antibiotics Reveals Illicit Transport of Kasugamycin and Blasticidin S
Source: PLoS Genet. 2016 Jun 29;12(6):e1006124. doi: 10.1371/journal.pgen.1006124 (PMC4927156; doi:10.1371/journal.pgen.1006124)
Supplement: S2 Table — (DOCX) [file pgen.1006124.s007.docx]

**S2 Table. Strains and plasmids used in this study.**

| Strain Number | Genotype | References |
| --- | --- | --- |
| CAG454 | MG1655 (*Escherichia coli* K-12 F^-^ λ^-^ *ilvG*^-^ *rfb-50* *rph-1*) | Lab Stock |
| CAG67776 | MG1655 Δ*opp::FRT* | This study |
| CAG67779 | MG1655 Δ*dpp::FRT* | This study |
| CAG67781 | MG1655 Δ*opp::FRT* Δ*dpp::FRT* | This study |
| CAG67774 | MG1655 Δ*oppA::FRT* | This study |
| CAG67777 | MG1655 Δ*dppA::FRT* | This study |
| CAG67780 | MG1655 Δ*oppA::FRT* Δ*dppA::FRT* | This study |
| CAG67775 | MG1655 Δ*oppB::FRT* | This study |
| CAG67778 | MG1655 Δ*dppB::FRT* | This study |
| CAG67788 | MG1655 Δ*oppB::FRT* Δ*dppB::FRT* | This study |
| CAG67789 | MG1655 Δ*gcvA::kan* | This study |
| CAG67790 | MG1655 Δ*gcvB::kan* | This study |
| CAG67791 | MG1655 Δ*opp::FRT* Δ*dpp::FRT* Δ*gcvA::kan* | This study |
| CAG67792 | MG1655 Δ*opp::FRT* Δ*dpp::FRT* Δ*gcvB::kan* | This study |
| pDSW204 | pTrc99a with promoter down mutation in -35 | [81] |
| pDSW204-opp | *oppABCDF* cloned into pDSW204 using BbsI and HindIII sites | This study |
| pBAD22 | - | [67] |
| pBAD22-oppA | oppA cloned into pBAD22 using NcoI and HindIII sites | This study |
| pKD4 | - | [82] |
| pCP20 | - | [82] |
