# Supplementary figures and images for: Correction: A Chemical-Genomic Screen of Neglected Antibiotics Reveals Illicit Transport of Kasugamycin and Blasticidin S
Source: PLoS Genet. 2017 Jul 21;13(7):e1006902. doi: 10.1371/journal.pgen.1006902 (PMC5521741; doi:10.1371/journal.pgen.1006902)

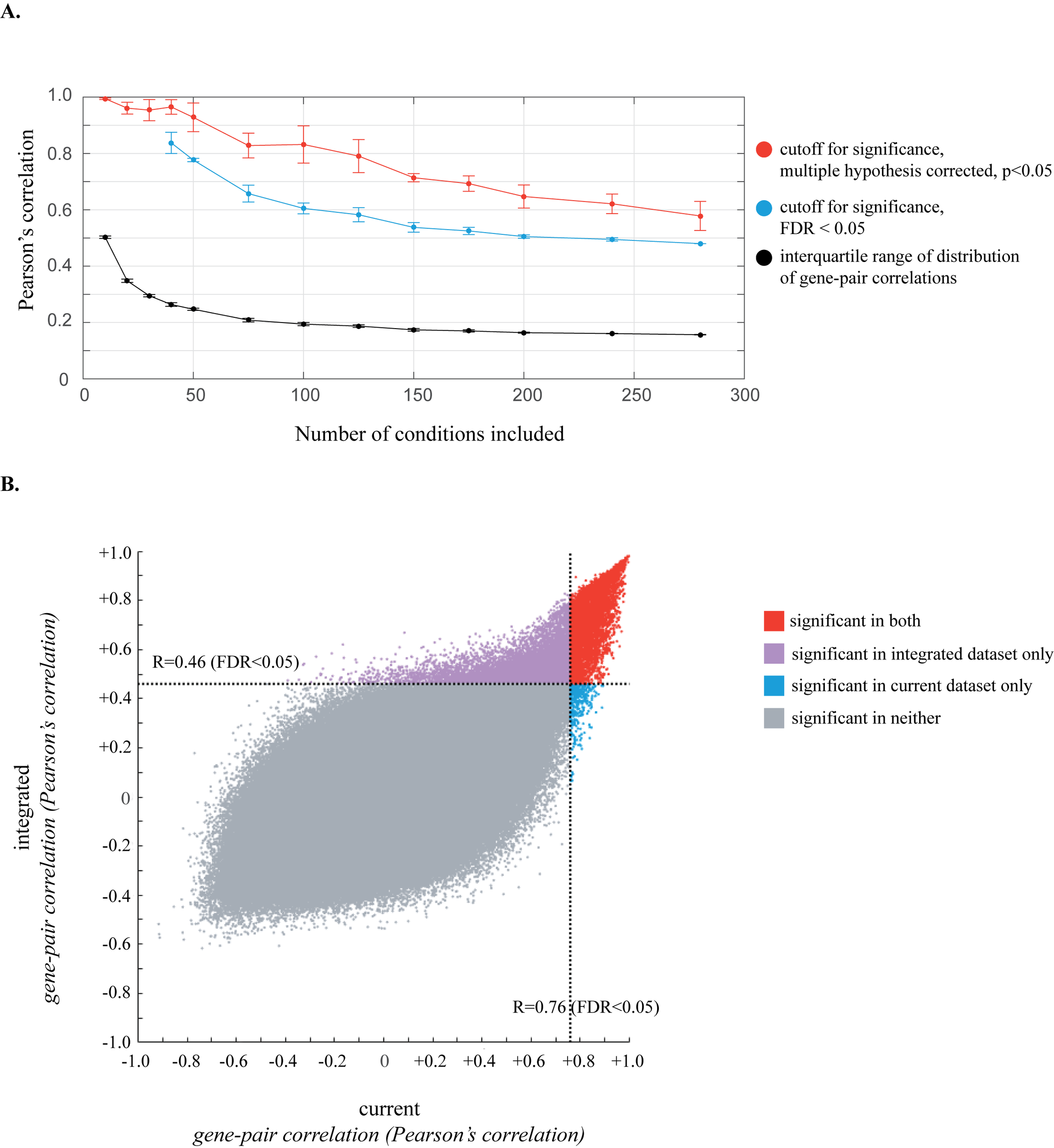

Supplement: S1 Fig — (A) More conditions increase the statistical significance of gene-pair correlations. The interquartile range of the distribution of gene-pair correlations, the cutoff for significance using a false discovery rate (FDR) of 5%, and the cutoff for a multiple hypothesis corrected p- value of 5% are plotted against number of conditions sampled from the Nichols et al. dataset [8]. Both methods for determining statistical significance are described in Nichols et al. [8]. Conditions were chosen randomly from the dataset in 4 independent samplings at each position, averages are plotted. Both variation and significance cutoffs decrease with an increasing number of conditions. The IQR (0.25) and FDR-based cutoff (0.76) of the current chemical-genomic screen (N = 51) are similar to a dataset of similar size sampled from Nichols et al. [8], indicating that differences in these statistical measures are due to dataset size only. (B) Integration of the current screen with a larger resource increases the number of statistically significant gene-pair correlations. In addition to reducing the variability of gene-pair correlations, integration of the current chemical-genomic screen with the larger dataset from Nichols et al. [8] lowered the cutoff for statistical significance from 0.76 to 0.46, including a larger fraction of the pairwise correlations between genes. (TIF) [file pgen.1006902.s001.tif]
