# Supplementary material for: Correction: A Chemical-Genomic Screen of Neglected Antibiotics Reveals Illicit Transport of Kasugamycin and Blasticidin S
Source: PLoS Genet. 2017 Jul 21;13(7):e1006902. doi: 10.1371/journal.pgen.1006902 (PMC5521741; doi:10.1371/journal.pgen.1006902)
Supplement: S1 Table — (DOCX) [file pgen.1006902.s003.docx]

**Supplementary Table 1: Cold-Sensitive Genes from the Screen**

| gene | COG category | Cold shock or translation reference | 10C sensitive | 10C fitness-score | 16C sensitive | 16C score |
| --- | --- | --- | --- | --- | --- | --- |
| istR-1 |  |  | TRUE | -16.4 | TRUE | -8.9 |
| typA | T | (1) | TRUE | -14.4 | TRUE | -7.6 |
| **ihfB** | **L** |  | TRUE | -12.1 | FALSE | -0.7 |
| **ihfA** | **L** |  | TRUE | -12.0 | FALSE | -2.0 |
| dinJ | V |  | TRUE | -11.5 | TRUE | -4.0 |
| ydfV |  |  | TRUE | -10.4 | FALSE | -1.0 |
| ymcE |  | (2) | TRUE | -10.3 | FALSE | -2.7 |
| **fusA-SPA** | **J** |  | TRUE | -9.8 | FALSE | -2.5 |
| **rbfA** | **J** | (3) | TRUE | -9.5 | FALSE | 4.0 |
| **deaD** | **L** | (4) | TRUE | -9.3 | TRUE | -9.5 |
| **dnaB-SPA** | **L** |  | TRUE | -9.2 | TRUE | -5.3 |
| smpB | O |  | TRUE | -9.0 | FALSE | -3.1 |
| dcrB |  |  | TRUE | -8.8 | FALSE | -1.3 |
| **dnaA-SPA** | **L** |  | TRUE | -8.6 | TRUE | -8.7 |
| yciM | M |  | TRUE | -8.5 | FALSE | -1.3 |
| hfq | T |  | TRUE | -7.9 | FALSE | -0.7 |
| **pheT-SPA** | **J** |  | TRUE | -7.2 | FALSE | 1.3 |
| crr | G |  | TRUE | -6.7 | FALSE | -2.0 |
| **yjgA** | **J** | (5) | TRUE | -6.4 | TRUE | -5.9 |
| spr | M |  | TRUE | -6.0 | FALSE | -0.2 |
| dnaJ | O |  | TRUE | -6.0 | TRUE | -3.4 |
| ptsN | GT |  | TRUE | -5.9 | TRUE | -3.8 |
| lipB | H |  | TRUE | -5.9 | FALSE | 3.8 |
| **frr-SPA** | **J** |  | TRUE | -5.8 | FALSE | 0.8 |
| glmS-SPA | M |  | TRUE | -5.7 | FALSE | -2.0 |
| ycbK | S |  | TRUE | -5.6 | FALSE | 0.1 |
| nfuA | O |  | TRUE | -5.6 | FALSE | -2.2 |
| der-SPA | R | (6) | TRUE | -5.4 | TRUE | -5.8 |
| rpsF |  | (7) | TRUE | -5.3 | FALSE | -1.8 |
| **rsmA** | **J** | (8) | TRUE | -5.2 | TRUE | -5.8 |
| sdhA | C |  | TRUE | -5.2 | FALSE | 0.2 |
| **rnhA** | **L** | (9) | TRUE | -5.1 | FALSE | -1.2 |
| **prfC** | **J** |  | TRUE | -5.0 | TRUE | -7.3 |
| **rluD** | **J** | (10) | TRUE | -4.9 | TRUE | -3.3 |
| aroK | E |  | TRUE | -4.9 | FALSE | -1.8 |
| gor | C |  | TRUE | -4.8 | FALSE | -1.2 |
| clpX | O |  | TRUE | -4.8 | FALSE | 5.5 |
| **rsmH** | **J** | (11) | TRUE | -4.8 | FALSE | 0.3 |
| ahpC | V |  | TRUE | -4.7 | FALSE | -3.0 |
| **dksA** | **J** |  | TRUE | -4.6 | FALSE | -0.5 |
| yjgZ |  |  | TRUE | -4.6 | FALSE | 2.0 |
| malT | K |  | TRUE | -4.6 | FALSE | 0.1 |
| **rsmB** | **JK** | (12) | TRUE | -4.5 | FALSE | 0.9 |
| ppk | P |  | TRUE | -4.5 | TRUE | -3.6 |
| cbrC | S |  | TRUE | -4.5 | FALSE | 1.0 |
| envC |  |  | TRUE | -4.4 | FALSE | 1.8 |
| wcaJ | M |  | TRUE | -4.3 | FALSE | 1.3 |
| yoaE | PR |  | TRUE | -4.3 | FALSE | 0.5 |
| fliQ | N |  | TRUE | -4.3 | FALSE | -0.4 |
| surA | O |  | TRUE | -4.2 | FALSE | 0.6 |
| gapA-SPA | G |  | TRUE | -4.1 | FALSE | -2.2 |
| yfdG | I |  | TRUE | -4.0 | TRUE | -4.4 |
| hipA | T |  | TRUE | -4.0 | FALSE | -0.6 |
| bamA{dup(218-219)} | M |  | TRUE | -3.9 | FALSE | 2.0 |
| kgtP | GEPR |  | TRUE | -3.9 | FALSE | -1.8 |
| fadR | K |  | TRUE | -3.9 | TRUE | -3.8 |
| **rpmE** | **J** |  | TRUE | -3.8 | TRUE | -4.8 |
| fliF | NU |  | TRUE | -3.8 | FALSE | 0.1 |
| mukE-SPA |  |  | TRUE | -3.8 | FALSE | 2.5 |
| waaF | M |  | TRUE | -3.8 | FALSE | 3.1 |
| grcA | H |  | TRUE | -3.8 | FALSE | 0.3 |
| cydB | C |  | TRUE | -3.8 | FALSE | 0.9 |
| **ybeY** | **J** |  | TRUE | -3.8 | FALSE | 2.1 |
| **dam** | **L** |  | TRUE | -3.8 | FALSE | 2.2 |
| **rpmG** | **J** |  | TRUE | -3.7 | FALSE | -2.8 |
| yhbJ | T |  | TRUE | -3.7 | FALSE | -0.7 |
| murD-SPA | M |  | TRUE | -3.7 | FALSE | -1.4 |
| ydfN |  |  | TRUE | -3.7 | FALSE | 0.5 |
| yaaI |  |  | TRUE | -3.6 | FALSE | -0.4 |
| yqeB | OR |  | TRUE | -3.6 | FALSE | -0.1 |
| cpxA | T |  | TRUE | -3.6 | FALSE | -2.7 |
| gpmM | G |  | TRUE | -3.6 | FALSE | 1.9 |
| dedD | D |  | TRUE | -3.5 | TRUE | -4.1 |
| yneJ | K |  | TRUE | -3.5 | FALSE | 1.4 |
| yejK | S |  | TRUE | -3.5 | FALSE | -0.3 |
| tolR | U |  | TRUE | -3.5 | FALSE | -0.4 |
| glgB | G |  | TRUE | -3.5 | FALSE | -2.8 |
| yggX | PO |  | TRUE | -3.5 | FALSE | -1.4 |
| pstS | P |  | TRUE | -3.5 | FALSE | 1.8 |
| pta | CR |  | TRUE | -3.4 | FALSE | -1.4 |
| murE-C | M |  | TRUE | -3.4 | FALSE | -0.6 |
| fbp | G |  | TRUE | -3.4 | FALSE | 4.8 |
| purR | K |  | TRUE | -3.4 | FALSE | -1.0 |
| rffM | M |  | TRUE | -3.4 | FALSE | -2.3 |
| apaH | T |  | TRUE | -3.4 | FALSE | -0.4 |
| ycjX | R |  | TRUE | -3.4 | FALSE | -0.9 |
| sdhB | C |  | TRUE | -3.4 | FALSE | 4.8 |
| rpiR | K |  | TRUE | -3.3 | FALSE | -1.7 |
| **seqA** | **L** |  | TRUE | -3.3 | FALSE | -0.7 |
| racC |  |  | TRUE | -3.3 | FALSE | -0.6 |
| gmm | F |  | TRUE | -3.3 | TRUE | -3.5 |
| yfiO* | M |  | TRUE | -3.3 | TRUE | -6.5 |
| pmbA | R |  | TRUE | -3.3 | FALSE | -1.6 |
| speB | E |  | TRUE | -3.2 | TRUE | -5.1 |
| rfe | M |  | TRUE | -3.2 | FALSE | -1.9 |
| ydfO | S |  | TRUE | -3.2 | FALSE | 0.4 |
| yejF | Q |  | TRUE | -3.2 | FALSE | -0.1 |
| rffE | M |  | TRUE | -3.2 | FALSE | 0.1 |
| rpoS | K |  | TRUE | -3.2 | FALSE | -1.7 |
| yhcO | K |  | TRUE | -3.1 | FALSE | -0.6 |
| ybjX | S |  | TRUE | -3.1 | FALSE | 1.1 |
| trxA | O |  | TRUE | -3.1 | FALSE | 2.3 |
| pstB | P |  | TRUE | -3.1 | FALSE | -0.3 |
| sixA | T |  | TRUE | -3.0 | FALSE | -0.5 |
| yfcP | N |  | TRUE | -3.0 | FALSE | -0.2 |
| rcsD | T |  | TRUE | -3.0 | FALSE | -1.0 |
| oppA | E |  | TRUE | -3.0 | FALSE | 0.4 |
| secB | U |  | FALSE | NaN | TRUE | -10.4 |
| yfcA | S |  | FALSE | NaN | TRUE | -6.3 |
| cpsG | G |  | FALSE | -0.2 | TRUE | -5.3 |
| fis | K |  | FALSE | 2.2 | TRUE | -5.1 |
| rnlB |  |  | FALSE | -1.2 | TRUE | -5.1 |
| yqjF | S |  | FALSE | 0.5 | TRUE | -5.1 |
| **rpsE-SPA** | **J** |  | FALSE | 0.1 | TRUE | -4.8 |
| yaiS | G |  | FALSE | -0.5 | TRUE | -4.5 |
| isrB |  |  | FALSE | -0.5 | TRUE | -4.3 |
| **infC-SPA** | **J** |  | FALSE | -2.3 | TRUE | -4.2 |
| dmsB | C |  | FALSE | 0.9 | TRUE | -4.0 |
| uup | R |  | FALSE | -1.8 | TRUE | -3.9 |
| **rbn** | **J** |  | FALSE | 1.0 | TRUE | -3.9 |
| ymiA |  |  | FALSE | 0.6 | TRUE | -3.8 |
| flhE |  |  | FALSE | -0.6 | TRUE | -3.8 |
| yegK |  |  | FALSE | NaN | TRUE | -3.8 |
| sbp | P |  | FALSE | 1.8 | TRUE | -3.7 |
| malS | G |  | FALSE | 0.3 | TRUE | -3.7 |
| gabT | E |  | FALSE | 1.3 | TRUE | -3.7 |
| rpoD-SPA | K |  | FALSE | 1.0 | TRUE | -3.7 |
| sgrT |  |  | FALSE | -1.8 | TRUE | -3.7 |
| fpr | C |  | FALSE | -0.8 | TRUE | -3.6 |
| yjfY |  |  | FALSE | -0.2 | TRUE | -3.6 |
| yehD | N |  | FALSE | 0.0 | TRUE | -3.6 |
| ycbC | R |  | FALSE | NaN | TRUE | -3.5 |
| fabF | IQ |  | FALSE | -2.3 | TRUE | -3.5 |
| **polB** | **L** |  | FALSE | -1.1 | TRUE | -3.5 |
| galE | M |  | FALSE | -1.1 | TRUE | -3.5 |
| ndk | F |  | FALSE | 0.3 | TRUE | -3.4 |
| hokD |  |  | FALSE | -1.2 | TRUE | -3.4 |
| sgcB | G |  | FALSE | 0.7 | TRUE | -3.4 |
| acpP-SPA | IQ |  | FALSE | 0.1 | TRUE | -3.3 |
| speE | E |  | FALSE | 0.9 | TRUE | -3.3 |
| ascG |  |  | FALSE | NaN | TRUE | -3.3 |
| ybhU |  |  | FALSE | -1.1 | TRUE | -3.2 |
| appY | K |  | FALSE | -2.1 | TRUE | -3.2 |
| **srmB** | **L** |  | FALSE | -1.2 | TRUE | -3.2 |
| hybE |  |  | FALSE | -0.7 | TRUE | -3.2 |
